# Targeting SLC1A5-mediated glutamine metabolism overcomes PARP inhibitor resistance in ovarian cancer

**DOI:** 10.1038/s41419-026-09123-z

**Published:** 2026-07-22

**Authors:** Fang Han, Hongjie Du, Kunlin Tian, Gonghua Qi, Beihua Kong, Xia Luo, Hanlin Ma

**Affiliations:** 1 Department of Obstetrics and Gynecology, Qilu Hospital of Shandong University, Jinan, China; 2 Shandong Key Laboratory of Reproductive Health and Birth Defects Prevention and Control, Qilu Hospital of Shandong University, Jinan, China; 3 Department of Ophthalmology, Qilu Hospital of Shandong University, Jinan, China; 4 Department of Otorhinolaryngology, Shandong Provincial Hospital Affiliated to Shandong First Medical University, Jinan, China

**Keywords:** Ovarian cancer, Cancer metabolism

## Abstract

Poly(ADP-ribose) polymerase inhibitors (PARPi), such as olaparib, have significantly improved outcomes in ovarian cancer (OC); however, therapy resistance remains a major clinical challenge. Here, we showed that glutamine metabolism was upregulated in PARPi-resistant OC cells and identified the glutamine transporter SLC1A5 as a key mediator of this metabolic adaptation. Inhibition of SLC1A5 increased the sensitivity of OC cells to olaparib both in vitro and in vivo. Mechanistically, we demonstrated that the deubiquitinating enzyme USP14 directly interacted with and stabilized SLC1A5 by removing K48-linked polyubiquitin chains, thereby enhancing glutamine uptake, promoting glutathione synthesis, alleviating oxidative stress, and activating the mTORC1 pathway. This metabolic rewiring improved DNA damage repair capacity and ultimately contributed to PARPi resistance. Notably, combined treatment with the SLC1A5 inhibitor V-9302 and olaparib synergistically suppressed tumor growth in a patient-derived xenograft (PDX) model. Collectively, our findings identify the USP14–SLC1A5 axis as a novel regulator of PARPi resistance and suggest that targeting glutamine metabolism represents a promising therapeutic strategy to overcome PARPi resistance in ovarian cancer.

**Figure:**
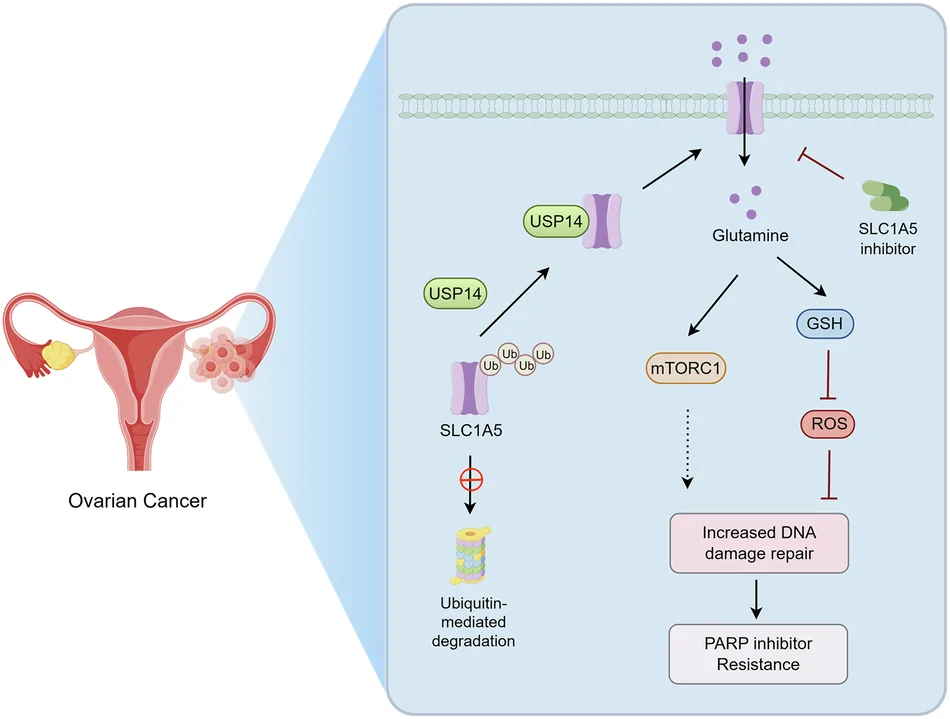

## Introduction

Ovarian cancer (OC) remains the most lethal gynecological malignancy, primarily due to its late‑stage diagnosis, frequent recurrence, and the development of therapeutic resistance. Epithelial OC accounts for approximately 90% of all OC cases, with high-grade serous ovarian cancer (HGSOC) representing nearly 70% of these cases [1]. Poly(ADP‑ribose) polymerase inhibitors (PARPi), such as olaparib, have significantly improved outcomes in patients with homologous recombination‑deficient (HRD) advanced OC [2]. However, patients with HRD-negative tumors derive limited benefit from PARPi therapy, and even those who initially respond often develop acquired resistance over time, ultimately leading to disease progression [3, 4]. Therefore, elucidating the mechanisms underlying PARPi resistance and developing effective strategies to overcome it are critical unmet needs in OC management.

Metabolic reprogramming is a well-established hallmark of cancer progression and therapeutic resistance. To meet the heightened biosynthetic and bioenergetic demands of rapid proliferation, cancer cells exhibit a pronounced dependence on glutamine [5]. Consequently, precise modulation of glutamine metabolism has emerged as a promising therapeutic strategy in cancer treatment. Beyond serving as a building block for protein synthesis, glutamine fuels the tricarboxylic acid (TCA) cycle, provides precursors for glutathione (GSH) synthesis and nucleotide production, and activates key pro‑growth signaling pathways such as mTORC1 [5, 6]. Although cells can synthesize glutamine de novo, proliferative demands often exceed endogenous production, necessitating robust extracellular uptake [7, 8]. The primary transporter responsible for this uptake is alanine-serine-cysteine transporter 2 (SLC1A5, also known as ASCT2) [9], which is frequently overexpressed in various tumors—including lung, head and neck, endometrial, and breast cancers—where its expression correlates with aggressive disease and poor prognosis [10–13]. Consequently, SLC1A5 represents a promising target for antitumor therapy. Clinical sample analyses reveal that SLC1A5 is overexpressed in OC tissues and correlates positively with poor prognosis [14]. Nevertheless, the precise role and regulatory mechanisms of SLC1A5 in OC PARPi resistance remain poorly understood.

Ubiquitination is a crucial post‑translational modification that regulates protein stability and function. Deubiquitinating enzymes (DUBs) counteract this process by removing ubiquitin chains, thereby stabilizing target proteins and preventing their proteasomal degradation [15]. Ubiquitin‑specific protease 14 (USP14), a key DUB family member, has been implicated in stabilizing oncoproteins, promoting tumor progression, and inducing chemoresistance in several cancers [16–21]. Previous studies indicate that USP14 is upregulated in OC and contributes to cisplatin resistance [22–24]. However, whether and how USP14 contributes to PARPi resistance, especially by modulating cancer metabolism such as glutamine utilization, remains unexplored.

In this study, we aimed to investigate the role of SLC1A5-mediated glutamine metabolism in PARPi resistance and to elucidate its upstream regulatory mechanisms. Using non-targeted metabolomics, we identified that glutamine metabolism was significantly upregulated in PARPi-resistant OC cells. Inhibition of the glutamine transporter SLC1A5 sensitized OC cells to olaparib in vitro and in a patient-derived xenograft (PDX) model. We further demonstrated that the deubiquitinase USP14 directly interacted with and stabilized SLC1A5 protein, thereby enhancing glutamine metabolism-related signaling pathways, which in turn improved DNA damage repair capacity and promoted PARPi resistance. Our findings establish the USP14–SLC1A5 axis as a novel driver of PARPi resistance and provide a compelling rationale for targeting SLC1A5 as a promising therapeutic strategy to restore PARPi sensitivity in OC.

## Materials and methods

### Cell lines and cell culture

The OC cell lines SKOV3 and OVCAR8 were obtained from the American Type Culture Collection (ATCC, Manassas, VA, USA), while the HEK293T cell line was procured from the Cell Bank of the Chinese Academy of Sciences (Shanghai, China). The olaparib-resistant SKOV3 (SK/Ola) cell line was established in our laboratory through continuous exposure of the parental SKOV3 cells to progressively higher doses of olaparib, following a previously reported protocol [25]. OVCAR8 cells were grown in RPMI 1640 medium (Gibco, Grand Island, NY, USA), whereas SKOV3 and SK/Ola cells were cultured in McCoy’s 5A medium (Gibco). HEK293T cells were maintained in high-glucose Dulbecco’s Modified Eagle Medium (DMEM, Gibco). All media were supplemented with 10% fetal bovine serum (Gibco) and 1% penicillin/streptomycin (15140-122, Gibco). To ensure quality control, all cell lines underwent routine mycoplasma contamination testing and were authenticated using short tandem repeat (STR) profiling. The cells were incubated at 37 °C in a humidified atmosphere containing 5% CO_2_.

### Antibodies and reagents

Antibodies targeting GST (10000-0-AP), His (66005-1-Ig), Flag (20543-1-AP), HA (81290-1-RR), GLS (12855-1-AP), GLUD1 (14299-1-AP), GLUL (11037-2-AP), α-tubulin (66031-1-Ig), SLC1A5 (20350-1-AP), 4EBP1 (60246-1-Ig), mTOR (66888-1-Ig), and USP14 (14517-1-AP) were procured from Proteintech (Wuhan, China). Antibodies against p-4EBP1 (2855) and p-mTOR (5536) were obtained from Cell Signaling Technology (Danvers, MA, USA). Antibody for β-actin (A5441) was obtained from Sigma-Aldrich (St. Louis, MO, USA). Ubiquitin (sc-8017) antibody was sourced from Santa Cruz Biotechnology (Dallas, TX, USA). The mouse IgG (A7028) and rabbit IgG (A7016) were purchased from Beyotime (Shanghai, China). Antibody against Ki-67 (ab245113), γH2AX (ab81299), H2AX (ab124781), and RAD51 (ab133534) were obtained from Abcam (Cambridge, UK). Chemical reagents such as olaparib (S1060), IU1-47 (S6943), V-9302 (S8818), glutamine (S1749), cycloheximide (CHX, S7418), and MG132 (S2619) were supplied by Selleck Chemicals (Houston, TX, USA).

### Western blot

Fresh frozen tissue samples from HGSOC patients and fallopian tubes were lysed by sonication in RIPA buffer (P0013B, Beyotime) supplemented with 1× Protease Inhibitor Cocktail (P1005, Beyotime). OC cells were washed with phosphate-buffered saline (PBS) and subsequently lysed using RIPA buffer supplemented with Protease Inhibitor Cocktail. Following centrifugation at 12,000 rpm for 5 min, the supernatants were collected. Protein concentrations were determined using the BCA Protein Assay Kit (P0012, Beyotime). Aliquots of protein lysates (20–40 µg) were separated by electrophoresis on 8–15% polyacrylamide gels and then transferred onto polyvinylidene fluoride (PVDF) membranes (Merck Millipore, Burlington, MA, USA). The membranes were blocked with 5% non-fat milk and sequentially probed with primary antibodies diluted at 1:1000 and horseradish peroxidase (HRP)-conjugated secondary antibodies diluted at 1:5000. Protein bands were detected using an enhanced chemiluminescence (ECL) detection kit (ORT2655, PerkinElmer, Waltham, MA, USA) and imaged with the GE Amersham Imager 600 (GE, Chicago, IL, USA). Band intensities were quantified using ImageJ software.

### Co-immunoprecipitation (Co-IP)

Cells were rinsed twice with PBS and lysed with Western and IP Lysis Buffer (P0013B, Beyotime) containing Protease Inhibitor Cocktail. The resulting cellular supernatants were collected after centrifugation. For immunoprecipitation (IP), 800 μg of the lysate was incubated overnight with 5 µL of primary antibodies or control rabbit/mouse IgG, followed by a 2-h incubation with protein A/G magnetic beads (B23202, Selleck) on a rotary shaker at 4 °C. The immune complexes were washed three times with Lysis Buffer and then denatured by boiling in 2× SDS loading buffer for subsequent western blot analysis. To minimize interference from denatured IgG, anti-HA Nanobody Magarose Beads (KTSM1335, AlpaLifeBio, Shenzhen, China), anti-Flag Beads (KTSM1338, AlpaLifeBio), or HRP-conjugated Mouse anti-Rabbit IgG Light Chain (AS061, ABclonal, Wuhan, China) were utilized.

### Mass spectrometry (MS) assay

SK/Ola cells stably expressing either control vector (Flag-tagged) or Flag-SLC1A5 were lysed using Western and IP Lysis Buffer. Following centrifugation, the clarified lysates were subjected to IP by incubating overnight with anti-Flag Beads. The beads were subsequently washed with lysis buffer to remove non-specific interactions, and the bound proteins were eluted by denaturation in SDS loading buffer. The immunoprecipitated proteins were then resolved by SDS-PAGE. Gel regions were carefully excised and prepared for further analysis. Liquid chromatography-tandem mass spectrometry (LC-MS/MS) was conducted by Qinglian Biotech Co., Ltd (Beijing, China) to identify the proteins. The list of SLC1A5-interacting proteins is presented in Supplementary Table 1.

### Immunohistochemistry (IHC) staining

HGSOC tissues were obtained from primary OC patients with no previous surgery or chemotherapy at Qilu Hospital of Shandong University. The clinicopathological characteristics of HGSOC samples are provided in Supplementary Table S2 (Supplementary information). Tumor tissues were harvested and promptly fixed in formalin solution overnight. The fixed tissues were then embedded in paraffin, sectioned into 4-µm-thick sections, and processed through deparaffinization and rehydration using a graded ethanol series. Antigen retrieval was performed using EDTA solution. Subsequently, the tissue sections were stained using a Universal Two Step Kit (PV9000, ZSGB-BIO, Beijing, China) following standard IHC protocols. Visualization was achieved using 3,3′-diaminobenzidine (DAB, ZSGB-BIO), and the sections were counterstained with hematoxylin. The stained slides were examined and imaged under an Olympus microscope (Tokyo, Japan). The expression levels in each sample were independently assessed by two pathologists, who evaluated both the staining intensity and the proportion of positive tumor cells. Staining intensity was graded as 0 (negative), 1 (weak), 2 (moderate), or 3 (strong), while the extent of staining was categorized as 1 (0–25%), 2 (26–50%), 3 (51–75%), or 4 (76–100%) based on the percentage of positively stained tumor cells. The final score was determined by multiplying the staining intensity by the extent of staining.

### Plasmid construction

The lentiviral overexpression constructs for USP14 and SLC1A5 were generated by cloning the open reading frames (ORFs) of human USP14 and SLC1A5 into the pCMV vector (PS100069, OriGene, Rockville, MD, USA). For gene knockdown experiments, shRNA sequences targeting USP14 and SLC1A5 were designed and cloned into the pLKO.1-puro vector (8453, Addgene, Watertown, MA, USA), resulting in the creation of USP14 shRNA-1, USP14 shRNA-2, SLC1A5 shRNA-1, and SLC1A5 shRNA-2 plasmids. The specific shRNA sequences are provided in Supplementary Table S3 (Supplementary information). Additionally, HA-USP14 and Flag-SLC1A5 were constructed by inserting their respective ORFs into pcDNA3.1 vectors (Addgene). All plasmid constructs were verified for accuracy through DNA sequencing.

### Transfection

For plasmid transfection, the Lipofectamine 2000 reagent (11668-019, Invitrogen) was utilized according to the manufacturer’s instructions. Lentivirus production was conducted in HEK293T cells by co-transfecting lentivectors with the packaging plasmids psPAX2 and pMD2.G. To generate stable cell lines, cells were infected with lentivirus for 24 h and then cultured in medium supplemented with 2 μg/mL puromycin (P8833, Sigma-Aldrich) for two weeks to select successfully transduced cells. The efficacy of gene knockdown and overexpression was confirmed through western blot analysis.

### Apoptosis assay

Apoptosis was assessed using the Annexin V APC Apoptosis Detection Kit (62700-80, BioGems, Westlake Village, CA, USA). In brief, cells were detached using trypsin, washed with PBS, and resuspended in 1× Annexin V Binding Buffer. Single-cell suspensions containing 1 × 10^6^ cells were incubated with 5 µL of Annexin V APC for 5 min and 5 µL of 7-AAD for 15 min on ice in the dark. The stained cells were analyzed using a BD FACSCalibur flow cytometer, and the resulting data were processed with FlowJo software.

### Colony formation assay

The single-cell suspensions were prepared and seeded at a density of 600 cells per well in 6-well plates. Following the specified treatments, the colonies were fixed with methanol for 15 min and subsequently stained with crystal violet for an additional 15 min. Colonies were imaged, and those containing >50 cells were counted using ImageJ.

### MTT assay

The cells were plated at a density of 3000 cells per well in 96-well plates. Following the designated treatments, 20 µL of MTT solution was added to each well, and the plates were incubated at 37 °C for 4 h. After incubation, the medium was carefully removed, and 200 µL of dimethyl sulfoxide (DMSO) was added to each well to dissolve the formazan crystals. The plates were gently vortexed in the dark for 10 min to ensure homogeneity. Absorbance at 490 nm was measured using a Thermo Scientific Microplate Reader (Waltham, MA, USA).

### Quantitative real-time PCR (qRT-PCR)

Total RNA was isolated using TRIzol reagent (15596018, Invitrogen) in accordance with the manufacturer’s protocol. Subsequently, 1 μg of total RNA was reverse transcribed into cDNA using the PrimeScript RT Reagent Kit (RR037A, TaKaRa, Kyoto, Japan). qRT-PCR was conducted using the SYBR Premix Ex Taq system (RR420A, TaKaRa) on a 7900HT Fast Real-Time PCR instrument (Applied Biosystems, Waltham, MA, USA). The relative mRNA expression levels of target genes were determined using the comparative Ct method (2^−ΔΔCt^), with β-actin as the internal reference gene. The primer sequences utilized are provided in Supplementary Table S4 (Supplementary information).

### GST pull-down assay

The coding sequences of USP14 and SLC1A5 were inserted into the pGEX-4T-1 and pET-22b(+) vectors, respectively, to construct GST-USP14 and His-SLC1A5 fusion proteins. These recombinant plasmids were transformed into E. coli BL21 (DE3), and protein expression was induced by adding 1 mM IPTG. The GST-USP14 protein was purified using a GST-tag Protein Purification Kit (P2262, Beyotime), while His-SLC1A5 was isolated using a His-Tag Protein Purification Kit (P2247, Beyotime). For the GST pull-down assay, GST-USP14, His-SLC1A5, and GST-tag Purification Resin (P2253, Beyotime) were co-incubated in binding buffer for 2 h. After incubation, the resin was washed twice with washing buffer, and the bound proteins were eluted by boiling in SDS loading buffer. The eluted proteins were then subjected to western blot analysis.

### In vitro de-ubiquitination (DUB) assay

HEK293T cells were co-transfected with Flag-SLC1A5 and HA-Ub plasmids for 48 h, followed by treatment with 10 μM MG132 for 6 h. IP was then performed using anti-Flag beads to isolate Flag-SLC1A5. Concurrently, His-USP14 was expressed and purified from E. coli BL21 (DE3). The purified His-USP14 protein was incubated with the Flag-SLC1A5-bound beads in DUB assay buffer (50 mM Tris HCl pH 7.5/1 mM EDTA/100 mM NaCl/0.05% CHAPS/5 mM DTT) for 2 h at 37 °C. After incubation, the mixture was denatured by boiling in SDS loading buffer and analyzed via western blot.

### Non-targeted metabolomics analysis

SKOV3 and SK/Ola cells were washed twice with cold PBS. After removing the PBS, five volumes of quenching reagent were added to halt metabolic activity, and cells were scraped using a cell scraper. The samples were centrifuged at 4 °C and 1000 × *g* for 1 min to remove the supernatant. Non-targeted metabolomics analysis was conducted and analyzed by Biomarker Technologies (Beijing, China).

### Animal experiments

Female BALB/c nude mice (4–6 weeks old) were purchased from Gempharmatech Co., Ltd (Nanjing, China) and housed under specific pathogen-free (SPF) conditions. A suspension of 5 × 10^6^ cells in PBS, either with or without SLC1A5 knockdown, was subcutaneously injected into the flanks of the mice. Once tumor volume reached approximately 50 mm^3^, the mice received intraperitoneal injections of olaparib (50 mg/kg) every other day. After three weeks of treatment, mice were euthanized by cervical dislocation, and xenograft tumors were collected for subsequent analysis. Tumor volume was calculated using the formula: volume = (length × width^2^) × 0.5.

For the PDX model, female NCG mice (4–6 weeks old) were obtained from Gempharmatech Co., Ltd and maintained under SPF conditions. Fresh tumor tissue was collected from consented patients, mechanically homogenized, and subcutaneously implanted into the lower dorsal flank of NCG mice. After stable engraftment, homogenized tumor tissue from the established PDX was passaged into new recipient NCG mice. Two weeks later, mice were randomly assigned to four treatment groups: (a) vehicle control, (b) olaparib, (c) V-9302, and (d) olaparib combined with V-9302. Mice received intraperitoneal injections of olaparib (50 mg/kg), V-9302 (50 mg/kg), or the drug combination every other day. Following four weeks of treatment, the mice were euthanized and subjected to further analysis.

### GSH measurements

OC cells were seeded in 6 cm dishes at a density of 5 × 10^5^ cells per dish. Following treatment, cells were harvested and intracellular glutathione (GSH) levels were quantified using a GSH/GSSG assay kit (S0053, Beyotime) according to the manufacturer’s protocol.

### ROS measurements

Cells were seeded in 96-well plates at a density of 2 × 10^4^ cells per well. After treatment, the culture medium was removed, and intracellular ROS levels were measured using the fluorescent probe DCFH-DA (ab113851, Abcam) according to the manufacturer’s instructions. Fluorescence intensity was measured using a Thermo Scientific Microplate Reader with excitation/emission wavelengths set at 485/535 nm.

### Glutamine uptake measurement

Glutamine (Gln) uptake was quantified using a glutamine assay kit (Abnova, KA1627) in accordance with the manufacturer’s guidelines. The uptake was determined by subtracting the measured glutamine concentration in the culture medium from the initial glutamine concentration. All obtained values were normalized to the corresponding cell number to ensure accurate comparisons.

### Immunofluorescence (IF) staining

Cells were plated in 15 mm glass-bottom culture dishes (NEST Biotech, Wuxi, China) and treated with olaparib. Following treatment, samples were processed through sequential washing with PBS, fixation in 4% paraformaldehyde (15 min), permeabilization with 0.2% Triton X-100 (5 min), and blocking with normal goat serum (30 min). IF staining was then performed by incubating with primary antibodies against RAD51 (1:100) or γH2AX (1:100) overnight, followed by Alexa Fluor-488-conjugated secondary antibody (1:200, A-11008) for 1 h in the dark. Nuclei were counterstained with DAPI, and imaging was conducted using a Sunny IRX-60 confocal microscope (Sunny Technology, Beijing, China).

### Molecular docking

The crystal structure of SLC1A5 was obtained from the RCSB PDB database (https://www.rcsb.org/structure/8OUD), while the structure of USP14 was predicted using AlphaFold 3. Both protein structures were processed with the Protein Preparation Wizard module in Schrödinger’s Maestro 14.3 through the following steps: protein preprocessing, regenerating states of native ligands, H-bond assignment optimization, protein energy minimization, and removal of water molecules. The prepared structures were then subjected to protein-protein interaction (PPI) simulations. The PPI complex with the lowest interaction score was visualized with distinct chains colored separately and surface representation added to display the 3D structure. Furthermore, the Protein Interaction Analysis module was employed to identify specific binding regions between SLC1A5 and USP14.

#### Comet assay

The Comet Assay was performed using Comet Assay Kit (4250-050-K, R&D Systems, MN, USA) in accordance with the manufacturer’s instructions. Briefly, following treatment, cells were harvested and resuspended in ice-cold PBS at a density of 1 × 10^5^ cells/mL. This suspension was mixed with pre-warmed (37 °C) LMAgarose at a 1:10 (v/v) ratio, and the mixture was immediately applied onto designated slides. Subsequent steps involved cell lysis, alkaline DNA unwinding, and electrophoresis under alkaline conditions. The DNA was then stained with SYBR Green I for 15 min under light-protected conditions. Comet images were visualized and acquired using a fluorescence microscope (X81, Olympus Optical, Tokyo, Japan).

### Statistical analysis

Data are expressed as mean ± standard error of the mean (SEM). Statistical analyses were conducted using GraphPad Software (La Jolla, CA, USA). Comparisons between two groups were performed using the Student’s *t* test, while comparisons involving three or more groups were analyzed by one-way analysis of variance (ANOVA). All experiments were replicated independently at least three times unless stated otherwise. A *p*-value of less than 0.05 (*p* < 0.05) was considered statistically significant. The correlation between USP14 and SLC1A5 was evaluated using Spearman’s correlation coefficient, calculated with GraphPad. The correlation between overall survival (OS) and SLC1A5 expression was analyzed using Kaplan–Meier survival analysis with the log-rank test. The combination effect between olaparib and V-9302 was calculated by CompuSyn software with the Chou–Talalay method. The following criteria were used to evaluate drug synergy: CI > 1 indicates antagonism, CI < 1 indicates synergy, CI between 0.3–0.7 indicates synergism, and CI between 0.1–0.3 indicates strong synergism. Clinical characteristics were analyzed using Chi-square test.

## Results

### SLC1A5 is associated with PARPi resistance in OC

**Fig. 1:**
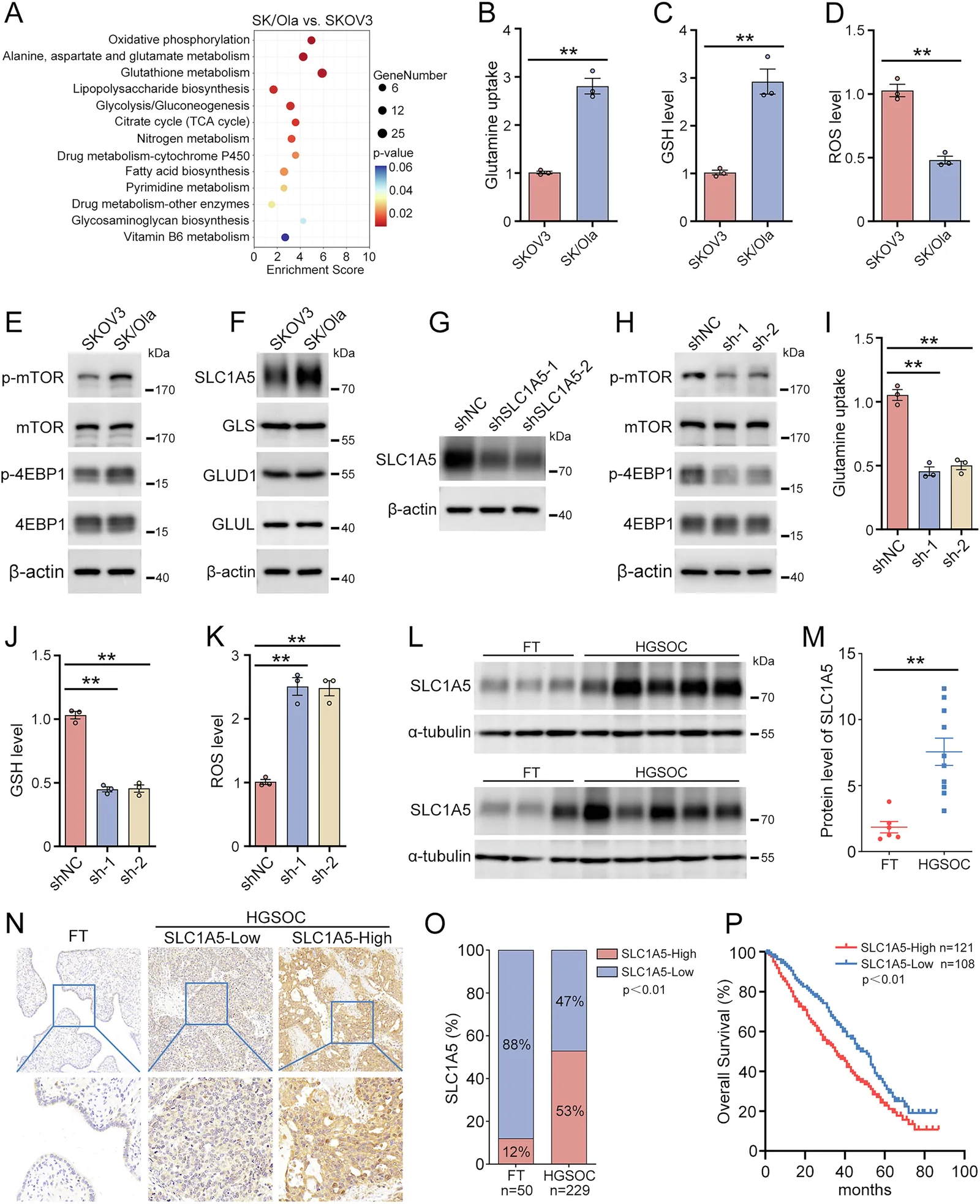
SLC1A5 is associated with PARPi resistance in OC. **A** Non-targeted metabolomic profiling was performed to identify differentially regulated metabolic pathways between olaparib-resistant SKOV3 (SK/Ola) and parental SKOV3 cells. **B** Glutamine uptake was measured in SK/Ola and SKOV3 cells. **C** Intracellular glutathione (GSH) levels were measured in SK/Ola and SKOV3 cells. **D** Intracellular reactive oxygen species (ROS) levels were determined in SK/Ola and SKOV3 cells. **E** Protein levels of p-mTOR, mTOR, p-4EBP1, and 4EBP1 in SK/Ola and SKOV3 cells were assessed by western blot. **F** Protein expression of SLC1A5, GLS, GLUD1, and GLUL in SK/Ola and SKOV3 cells was analyzed by western blot. **G** SK/Ola cells were stably transduced with pLKO.1 control vector (shNC) or two independent SLC1A5 shRNAs (sh-1 and sh-2). Knockdown efficiency was verified by western blot analysis. **H** Following SLC1A5 knockdown, the protein levels of p-mTOR, mTOR, p-4EBP1, and 4EBP1 were examined by western blot in SK/Ola cells. **I** Glutamine uptake was measured following SLC1A5 knockdown in SK/Ola cells. **J** Intracellular GSH levels were measured following SLC1A5 knockdown in SK/Ola cells. **K** Intracellular ROS levels were measured following SLC1A5 knockdown in SK/Ola cells. **L** SLC1A5 protein expression in fallopian tube (FT) and high-grade serous ovarian carcinoma (HGSOC) tissues was evaluated by western blot. **M** Quantification of SLC1A5 protein expression shown in (**L**). **N** Representative immunohistochemical (IHC) staining images showing SLC1A5 expression in HGSOC and FT tissues (Upper panel, 40×; Lower panel, 400×). Positive SLC1A5 staining was observed in both tumor cells and stromal cells within the tumor microenvironment. **O** Distribution of SLC1A5 expression in 50 FT tissues and 229 HGSOC tissues. Cases were stratified into high- and low-SLC1A5 expression groups according to the IHC scoring criteria, and the proportion of samples in each group is shown. **P** Kaplan–Meier analysis of overall survival (OS) in patients with HGSOC. Patients were stratified into high- and low-SLC1A5 expression groups, and survival differences were assessed using the log-rank test. Quantification of protein levels shown in (**E**–**H**) is provided in Supplementary Fig. S1C–F. Data are expressed as the mean ± SEM (**p < 0.01, *n* = 3).

To identify key metabolic pathways involved in PARPi resistance, we performed non-targeted metabolomic analysis on olaparib-resistant SKOV3 cells (SK/Ola) and their parental SKOV3 cells. Glutamine metabolism-related pathways emerged as the most significantly altered pathways between the two cell lines (Fig. 1A, Supplementary Fig. S1A). Subsequent validation experiments confirmed that, compared to parental SKOV3 cells, SK/Ola cells exhibited increased glutamine uptake and elevated intracellular glutathione (GSH) levels, accompanied by reduced ROS production (Fig. 1B–D). OVCAR8 and SKOV3 cells also showed a consistent increase in glutamine uptake under olaparib treatment (Supplementary Fig. S1B). Additionally, SK/Ola cells showed enhanced activation of the mTORC1 signaling pathway (Fig. 1E). We next examined the expression of key regulators involved in glutamine metabolism. Among these, only SLC1A5 expression was significantly altered in SK/Ola cells, while other factors remained unchanged (Fig. 1F), suggesting that SLC1A5 may be a central regulator of glutamine metabolism in OC. Knockdown of SLC1A5 (Fig. 1G) markedly suppressed mTORC1 activity, reduced glutamine uptake and GSH levels, and increased ROS accumulation (Fig. 1H–K) in SK/Ola cells. Although *SLC1A5* mRNA levels did not differ significantly between high-grade serous ovarian carcinoma (HGSOC) and fallopian tube (FT) tissues (Supplementary Fig. S1G, H), SLC1A5 protein expression was significantly upregulated in HGSOC samples (Fig. 1L, M). IHC analysis further revealed strong SLC1A5 staining in HGSOC tissues compared to FT controls. Quantification indicated that 53% of HGSOC samples were classified as SLC1A5-high, compared with only 12% of FT samples (Fig. 1N, O). Notably, SLC1A5 expression was not restricted to tumor cells; positive staining was also observed in stromal cells. This pattern is consistent with the known role of SLC1A5 in supporting the metabolic demands of various cell types within the tumor niche, including cancer-associated fibroblasts and infiltrating immune cells [26–28]. Clinically, HGSOC patients with high SLC1A5 expression had significantly shorter overall survival (OS) than those with low expression (Fig. 1P). Together, these findings indicate that SLC1A5 is associated with PARPi resistance in OC.

### SLC1A5 inhibition overcomes PARPi resistance in OC

**Fig. 2:**
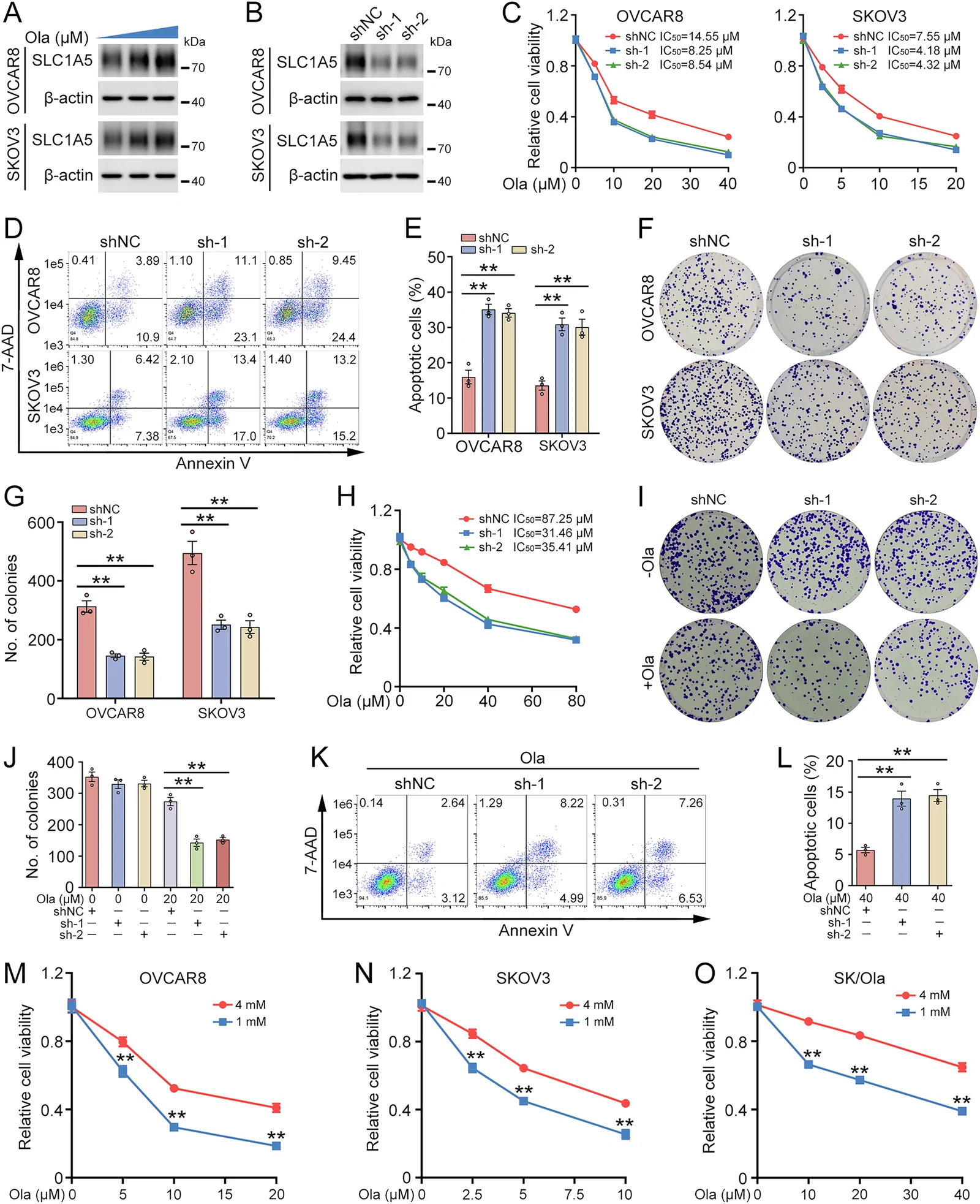
Knockdown of SLC1A5 sensitizes OC cells to olaparib. **A** Cells were treated with olaparib (OVCAR8: 10, 20 μM; SKOV3: 5, 10 μM) for 48 h, and SLC1A5 protein levels were evaluated by western blot. Quantification is provided in Supplementary Fig. S2B. **B** OVCAR8 and SKOV3 cells were stably transduced with a pLKO.1 control vector (shNC) or two independent SLC1A5-targeting shRNAs (sh-1 and sh-2). Knockdown efficiency was confirmed by western blot analysis. Quantification of SLC1A5 protein expression is provided in Supplementary Fig. S2E. **C** Cells were treated with increasing concentrations of olaparib for 72 h (OVCAR8: 5, 10, 20, and 40 μM; SKOV3: 2.5, 5, 10, and 20 μM), and cell viability was assessed by MTT assay. **D** Representative flow cytometry plots showing apoptosis in OVCAR8 and SKOV3 cells following treatment with olaparib (OVCAR8: 20 μM; SKOV3: 10 μM) for 72 h. **E** Quantification of apoptotic cells shown in (**D**). **F** Colony formation assays were performed in cells treated with olaparib (OVCAR8: 10 μM; SKOV3: 5 μM). **G** Quantification of colony numbers shown in (**F**). **H** Cell viability was assessed using the MTT assay in SK/Ola cells treated with increasing concentrations of olaparib (5, 10, 20, 40, 80 μM) for 72 h. **I** Colony formation assay was employed in SK/Ola cells treated with 20 μM olaparib. **J** Quantification of colony numbers shown in (**I**). **K** Representative flow cytometry plots showing apoptosis in SK/Ola cells following treatment with 40 μM olaparib for 72 h. **L** Quantification of apoptotic cells shown in (**K**). **M**–**O** OVCAR8, SKOV3, and SK/Ola cells were cultured in medium containing either 4 mM (high) or 1 mM (low) glutamine and treated with the indicated concentrations of olaparib for 72 h. Cell viability was subsequently evaluated using the MTT assay. Data are expressed as the mean ± SEM (*p < 0.05, **p < 0.01, *n* = 3).

First, we examined the effect of olaparib on SLC1A5 expression in OC cells. Olaparib treatment significantly increased SLC1A5 protein levels without affecting its mRNA expression (Fig. 2A and Supplementary Fig. S2A), suggesting post-translational regulation. To directly test whether olaparib enhances SLC1A5 protein stability, we performed cycloheximide (CHX) chase assays. As shown in Supplementary Fig. S2C, D, SLC1A5 protein degraded more slowly in olaparib-pretreated cells compared to control cells, with a markedly prolonged half-life. To further clarify the role of SLC1A5 in olaparib sensitivity, we generated lentivirus-mediated SLC1A5 knockdown cells (Fig. 2B). MTT assays demonstrated that SLC1A5 knockdown significantly increased the sensitivity of OC cells to olaparib, as reflected by reduced cell viability (Fig. 2C). This finding was consistently validated by flow cytometry, which revealed elevated apoptosis rates (Fig. 2D, E), and by colony formation assays, which showed decreased clonogenic capacity in cells with SLC1A5 knockdown (Fig. 2F, G). Next, we evaluated the effect of SLC1A5 on olaparib sensitivity in olaparib-resistant SK/Ola cells. While SK/Ola cells exhibited limited response to olaparib, SLC1A5 knockdown restored olaparib sensitivity (Fig. 2H). Additionally, SLC1A5 knockdown reduced the colony-forming ability of SK/Ola cells under olaparib treatment (Fig. 2I, J) and enhanced olaparib-induced apoptosis compared to controls (Fig. 2K, L). Furthermore, to assess the impact of glutamine availability on PARPi sensitivity, we treated OC cells with olaparib under varying glutamine concentrations. MTT assays demonstrated that higher glutamine levels markedly enhanced cell survival during olaparib exposure, whereas glutamine restriction sensitized cells to PARPi (Fig. 2M–O). Together, these results indicate that SLC1A5 acts as a key regulator promoting olaparib resistance in OC.

### SLC1A5 inhibitor V-9302 sensitizes OC cells to olaparib

**Fig. 3:**
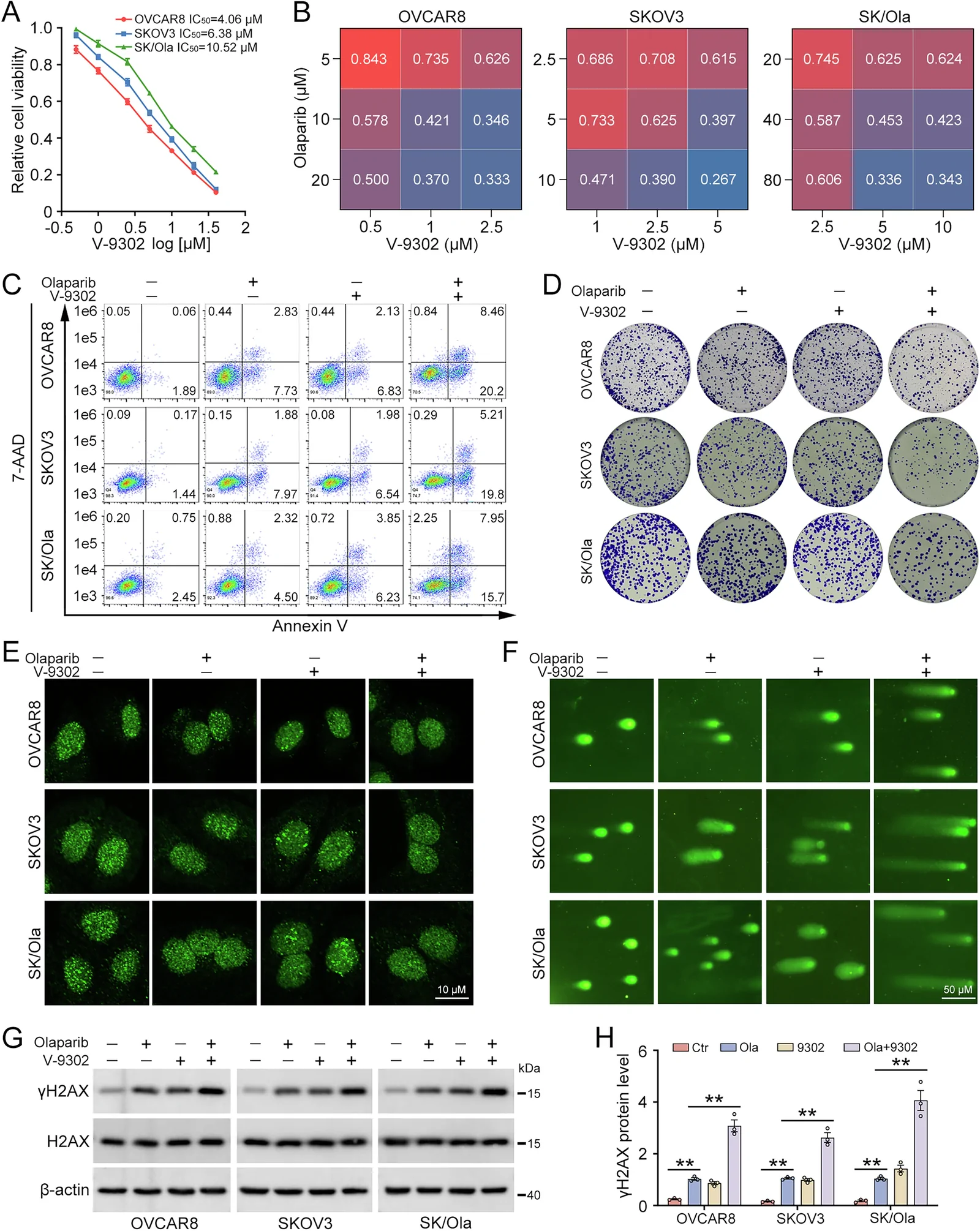
Inhibition of SLC1A5 by V-9302 overcomes olaparib resistance in OC cells. **A** The half-maximal inhibitory concentration (IC_50_) values of the SLC1A5 inhibitor V-9302 in OVCAR8, SKOV3, and SK/Ola cells were determined using MTT assay. **B** Synergistic effects between V-9302 and olaparib were evaluated by calculating the Combination Index using CompuSyn software. **C** Representative flow cytometry plots showing apoptosis in OVCAR8, SKOV3, and SK/Ola cells treated with V-9302 and/or olaparib for 72 h. Quantification of apoptotic cells is provided in Supplementary Fig. S3A. **D** Colony formation assays were performed in cells treated with V-9302 and/or olaparib. Quantification of colony numbers is provided in Supplementary Fig. S3B. **E** Representative immunofluorescence images of RAD51 foci in cells treated with V-9302 and/or olaparib for 48 h. Scale bar: 10 µm. Quantification of RAD51 foci is provided in Supplementary Fig. S3C. **F** Representative comet assay images of cells treated with V-9302 and/or olaparib for 48 h. Scale bar: 50 µm. Quantification of tail moment is provided in Supplementary Fig. S3D. **G** The protein levels of γH2AX and H2AX were analyzed using western blot in cells treated with V-9302 and/or olaparib for 48 h. **H** Quantification of γH2AX protein levels shown in (**G**). Data are expressed as the mean ± SEM (*p < 0.05, **p < 0.01, *n* = 3).

V-9302, a widely used inhibitor of SLC1A5, competitively antagonizes glutamine uptake and has been employed in multiple studies targeting glutamine metabolism [29]. We next evaluated the potential synergistic effect of V-9302 and olaparib in OC cells. First, we determined the IC_50_ values of V-9302 using the MTT assay, which yielded values of 4.06 μM in OVCAR8, 6.38 μM in SKOV3, and 10.52 μM in SK/Ola cells (Fig. 3A). The Chou–Talalay analysis indicated that V-9302 and olaparib synergistically inhibited OC cell growth across a range of concentrations, suggesting that V-9302 may act as an olaparib sensitizer (Fig. 3B). Flow cytometry analysis further revealed that the combination of V-9302 and olaparib significantly increased the proportion of apoptotic cells compared to either agent alone (Fig. 3C). Similarly, colony formation assays demonstrated that the combination treatment markedly reduced colony numbers relative to monotherapy (Fig. 3D). We then examined whether the combination treatment affects olaparib-induced DNA damage. IF staining of RAD51 foci showed that V-9302 combined with olaparib led to impaired DNA repair capacity compared to single treatments (Fig. 3E). The Comet Assay further confirmed that the combination treatment significantly exacerbated DNA damage compared to monotherapy (Fig. 3F). Consistently, western blot analysis showed increased γH2AX levels following the combination treatment (Fig. 3G, H). Collectively, these results demonstrate that V-9302 enhances the sensitivity of OC cells to olaparib by aggravating DNA damage, supporting its potential as an adjuvant to improve PARPi efficacy.

### USP14 directly interacts with SLC1A5

**Fig. 4:**
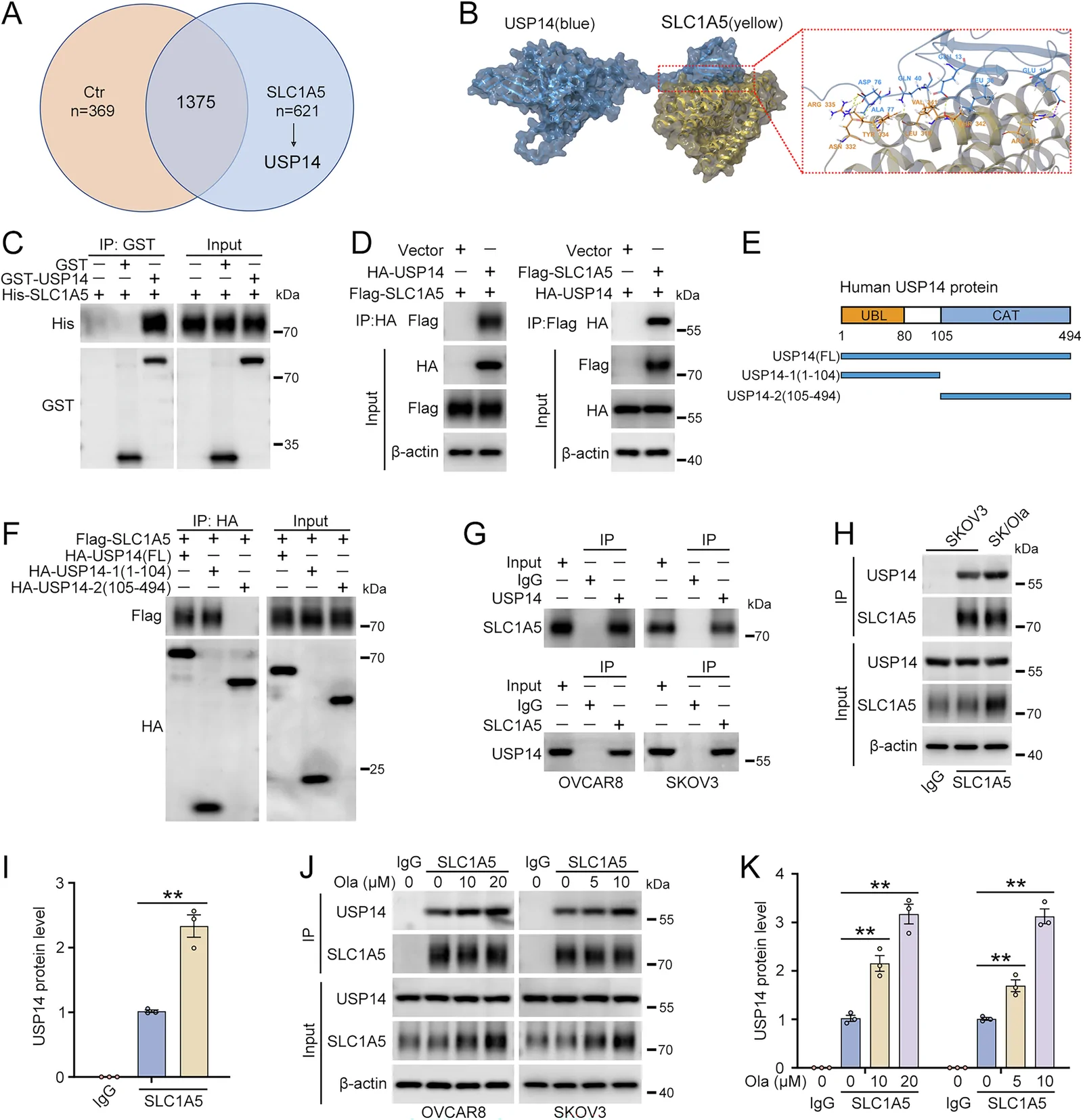
USP14 directly interacts with SLC1A5. **A** SLC1A5-interacting proteins were identified by immunoprecipitation (IP) followed by mass spectrometry analysis, with IgG serving as a negative control. A complete list of candidate SLC1A5-interacting proteins is provided in Supplementary Table 1. **B** Molecular docking model illustrating the predicted interaction between USP14 (blue) and SLC1A5 (yellow). The UBL domain of USP14 was predicted to mediate binding to SLC1A5. **C** GST pull-down assays were performed using purified GST-USP14 and His-SLC1A5 proteins. Proteins were incubated with GST resin in binding buffer for 2 h, followed by washing, elution, and western blot analysis. **D** HA-USP14 and Flag-SLC1A5 were co-transfected into HEK293T cells for 48 h, followed by co-immunoprecipitation (Co-IP) assay using anti-Flag or anti-HA beads. Co-immunoprecipitated proteins were detected using the indicated antibodies. **E** Schematic representation of full-length (FL) and truncated USP14 constructs used for interaction domain mapping. **F** FL and truncated HA-tagged USP14 constructs were co-transfected with Flag-SLC1A5 into HEK293T cells for 48 h. Co-IP assays were performed using anti-HA beads, and co-immunoprecipitated Flag-SLC1A5 was detected using an anti-Flag antibody. **G** Endogenous interaction between USP14 and SLC1A5 in OVCAR8 and SKOV3 cells was analyzed by Co-IP. **H** Co-IP assays were performed using an anti-SLC1A5 antibody in SK/Ola and SKOV3 cells, followed by western blot analysis with the indicated antibodies. **I** Quantification of the immunoprecipitated USP14 protein levels from (**H**). **J** Cells were treated with olaparib for 48 h, followed by immunoprecipitation using an anti-SLC1A5 antibody and immunoblotting with the indicated antibodies. **K** Quantification of the immunoprecipitated USP14 protein levels from (**J**). Data are expressed as the mean ± SEM (**p < 0.01, *n* = 3).

We next investigated the mechanism underlying the elevated expression of SLC1A5 in olaparib-resistant OC. Using SLC1A5 as bait in mass spectrometry analysis, we identified 621 SLC1A5-interacting proteins (Fig. 4A). Since olaparib treatment increased SLC1A5 protein expression without altering its mRNA level, we focused on interactors potentially involved in regulating protein stability. Among these candidates, the deubiquitinase USP14 scored highest and was therefore selected for further investigation (Supplementary Table 1). To verify this interaction, we performed molecular docking via Schrödinger’s Maestro calculations, which showed that the UBL domain of USP14 was critical for binding to SLC1A5 (Fig. 4B). Subsequently, purified GST-USP14 and His-SLC1A5 proteins were subjected to a GST pull-down assay, which confirmed their direct interaction (Fig. 4C). To further validate this interaction, HA-USP14 and Flag-SLC1A5 were co-transfected into HEK293T cells, followed by immunoprecipitation with anti-Flag or anti-HA antibodies, respectively, providing additional evidence of their association (Fig. 4D). To determine the specific domains of USP14 responsible for its interaction with SLC1A5, we generated and assessed various truncation mutants of USP14 for their binding capacity. Consistent with the prediction from molecular docking, the UBL domain of USP14 is essential for this interaction, whereas other domains are dispensable (Fig. 4E, F). The endogenous interaction between USP14 and SLC1A5 was further validated in OVCAR8 and SKOV3 cells (Fig. 4G). To investigate the effect of PARPi on this interaction, cells were treated with olaparib for 48 h. Co-IP results revealed that olaparib treatment strengthened the USP14-SLC1A5 interaction (Fig. 4J, K). Enhanced binding between USP14 and SLC1A5 was also observed in olaparib-resistant SK/Ola cells compared with parental SKOV3 cells (Fig. 4H, I). Collectively, these findings demonstrate a direct interaction between USP14 and SLC1A5, which is augmented by PARPi treatment.

### USP14 modulates the expression and stability of the SLC1A5 protein

**Fig. 5:**
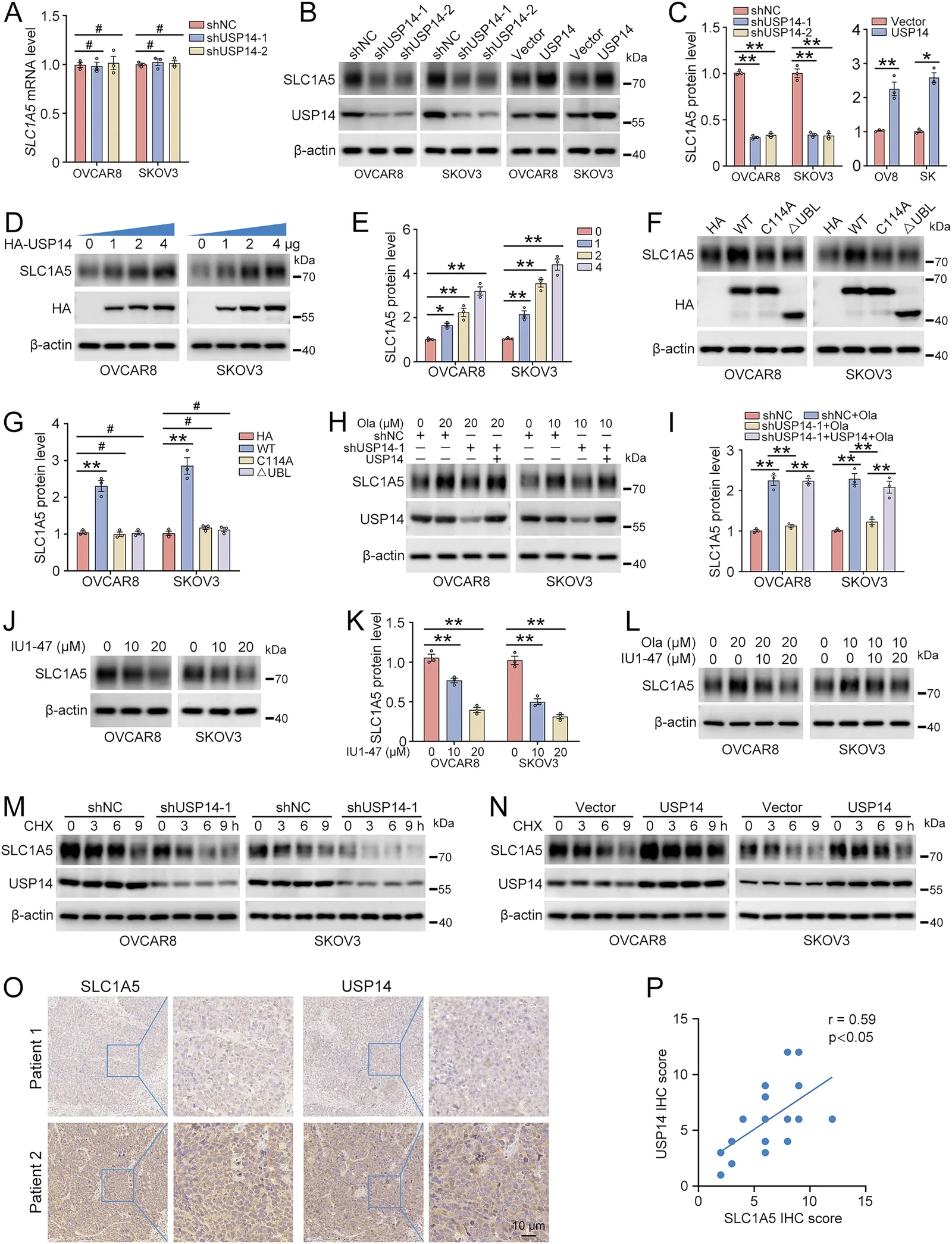
USP14 regulates the expression and stability of SLC1A5 protein. **A** OVCAR8 and SKOV3 cells were stably transduced with pLKO.1 control vector (shNC) or two independent USP14 shRNAs (shUSP14-1 and shUSP14-2). qPCR analysis was performed to assess SLC1A5 mRNA expression following USP14 knockdown. **B** OVCAR8 and SKOV3 cells were subjected to USP14 knockdown or USP14 overexpression using the indicated constructs. Protein levels of USP14 and SLC1A5 were analyzed by western blot following USP14 knockdown or overexpression. **C** Quantification of SLC1A5 protein levels shown in (**B**). **D** Cells were transiently transfected with increasing amounts of HA-USP14 plasmids (0, 1, 2, 4 µg) for 48 h, followed by western blot analysis of SLC1A5 and HA-USP14 protein levels. **E** Quantification of SLC1A5 protein levels shown in (**D**). **F** Cells were transiently transfected with empty vector (HA), HA-USP14 (WT), HA-USP14^C114A^ (C114A), or HA-USP14^ΔUBL^ (ΔUBL) for 48 h, followed by western blot analysis of SLC1A5 and USP14 protein levels. **G** Quantification of SLC1A5 protein levels shown in (**F**). **H** Cells with USP14 knockdown and/or USP14 overexpression were treated with olaparib for 48 h. Protein levels of USP14 and SLC1A5 were examined by western blot. **I** Quantification of SLC1A5 protein levels shown in (**H**). **J** Cells were treated with the USP14 inhibitor IU1-47 for 48 h, and SLC1A5 protein levels were assessed by western blot. **K** Quantification of SLC1A5 protein levels shown in (**J**). **L** Cells were treated with the indicated concentrations of IU1-47 and/or olaparib for 48 h. SLC1A5 protein levels were analyzed by western blot. Quantification is provided in Supplementary Fig. S4. **M** Protein stability of SLC1A5 was analyzed in USP14-knockdown cells. Cells were treated with cycloheximide (CHX, 100 μg/mL) for the indicated time periods, and SLC1A5 protein degradation was evaluated by western blot. **N** Protein stability of SLC1A5 was analyzed in USP14-overexpressing cells. Cells were treated with cycloheximide (CHX, 100 μg/mL) for the indicated times, followed by western blot analysis. **O** Representative IHC staining images showing USP14 and SLC1A5 expression in HGSOC tissues. Scale bar: 10 µm. **P** Correlation analysis between USP14 and SLC1A5 expression levels in HGSOC tissues based on IHC staining scores. Data are expressed as the mean ± SEM (^#^*p* > 0.05, *p < 0.05, **p < 0.01, *n* = 3).

First, we assessed whether USP14 regulated SLC1A5 expression. Knockdown of USP14 did not affect *SLC1A5* mRNA levels (Fig. 5A) but reduced SLC1A5 protein levels (Fig. 5B, C). Conversely, USP14 overexpression elevated SLC1A5 protein levels in a dose-dependent manner (Fig. 5B–E). However, neither the catalytically inactive mutant (USP14^C114A^) nor the ΔUBL truncation mutant (USP14^ΔUBL^) increased SLC1A5 expression (Fig. 5F, G). We then examined the effect of USP14 on SLC1A5 protein levels under olaparib treatment. Olaparib exposure increased SLC1A5 protein levels in cells expressing USP14, whereas knockdown of USP14 markedly reduced SLC1A5 levels. Importantly, this reduction was reversed by reintroduction of USP14, confirming the specificity of the effect (Fig. 5H, I). Furthermore, treatment with the USP14-specific inhibitor IU1-47 decreased SLC1A5 protein levels in a dose-dependent manner (Fig. 5J, K). Notably, IU1-47 also attenuated the olaparib-induced upregulation of SLC1A5 (Fig. 5L). We next examined whether USP14 modulates SLC1A5 expression by regulating its protein stability. As shown in CHX chase assays (Fig. 5M, N), depletion of USP14 accelerated SLC1A5 degradation, resulting in a shortened half-life. In contrast, USP14 overexpression decelerated its turnover, leading to a prolonged half-life. Additionally, IHC staining of USP14 and SLC1A5 in HGSOC tissues from our cohort revealed a positive correlation between their protein levels (Fig. 5O, P). Collectively, these results suggest that USP14 enhances the expression and stability of SLC1A5 in OC.

### USP14 regulates the ubiquitination of SLC1A5 as a deubiquitinase

We further investigated whether USP14 serves as a deubiquitinase for SLC1A5. Knockdown of USP14 elevated the ubiquitin levels in SLC1A5 immunoprecipitates (Supplementary Fig. S5A), whereas overexpression of wild-type USP14, but not its catalytically inactive form or ΔUBL truncation mutant, reduced SLC1A5 ubiquitination (Supplementary Fig. S5A, F). This indicates that the catalytic activity of USP14 is critical for SLC1A5 deubiquitination. To determine the specific polyubiquitin chains targeted by USP14, we co-transfected cells with His-Ub (K48-only or K63-only), HA-USP14, and Flag-SLC1A5. Results showed that USP14 selectively decreased K48-linked ubiquitination of SLC1A5, with no effect on K63-linked chains (Supplementary Fig. S5B). Additionally, USP14 overexpression failed to reduce SLC1A5 ubiquitination in cells expressing the K48R mutant (Lys48 mutated to Arg) (Supplementary Fig. S5C). Consistent with our prior observation that olaparib strengthened the USP14-SLC1A5 interaction, we found that olaparib treatment also diminished SLC1A5 ubiquitination (Supplementary Fig. S5E). The USP14-specific inhibitor IU1-47 increased SLC1A5 ubiquitination in a dose-dependent manner (Supplementary Fig. S5D) and counteracted the reduction in SLC1A5 ubiquitination induced by olaparib (Supplementary Fig. S5E). An in vitro deubiquitination assay further confirmed that purified USP14 efficiently removed ubiquitin from SLC1A5 (Supplementary Fig. S5G). Together, these findings indicate that USP14 protects SLC1A5 from degradation by selectively removing K48-linked polyubiquitin chains.

### USP14 contributes to the regulation of SLC1A5-dependent glutamine metabolic signaling

**Fig. 6:**
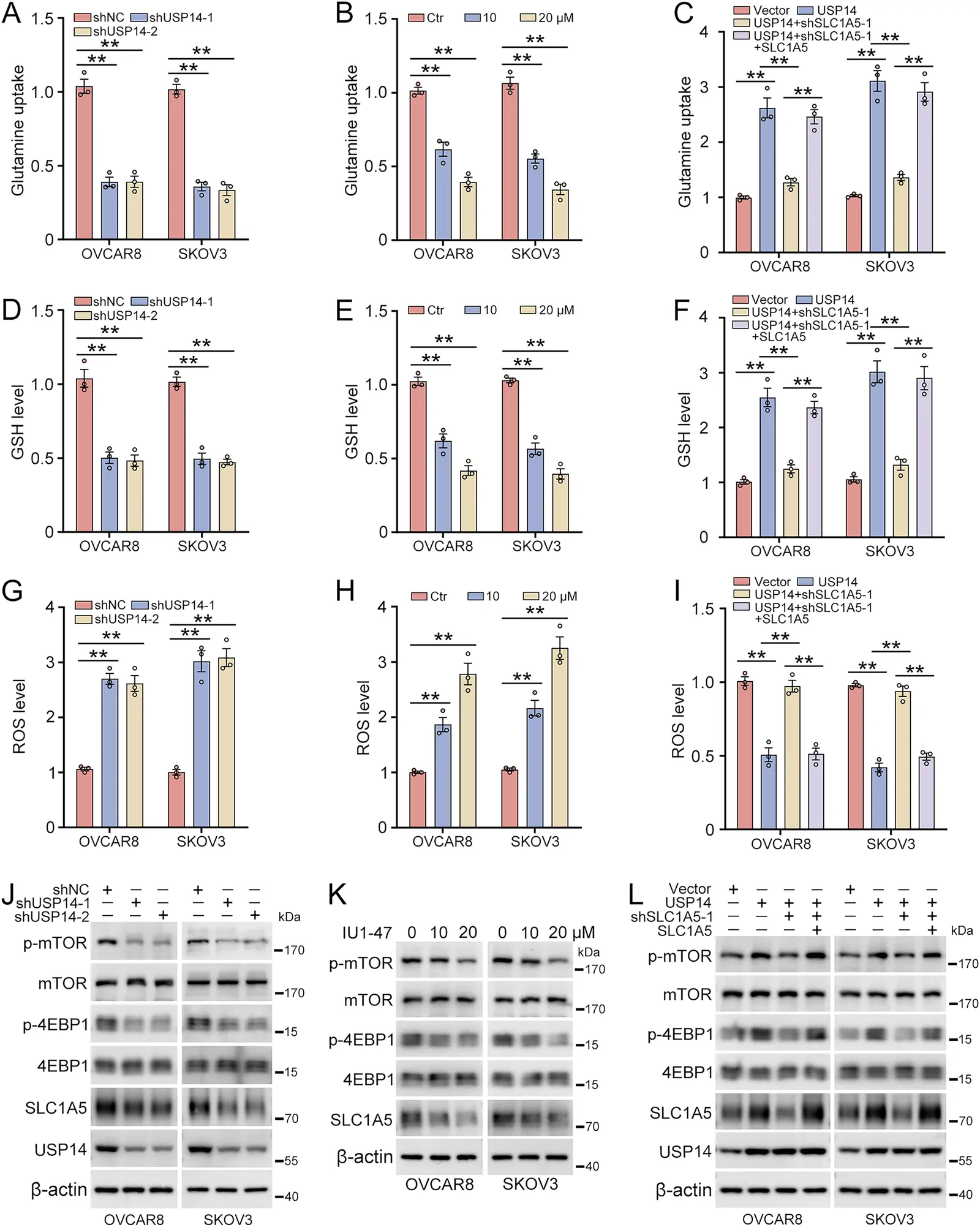
USP14/SLC1A5 regulates glutamine metabolism-related signaling pathways. **A** OVCAR8 and SKOV3 cells were stably transduced with pLKO.1 control vector (shNC) or two independent USP14 shRNAs (shUSP14-1 and shUSP14-2), and glutamine uptake was measured. **B** Cells were treated with the USP14 inhibitor IU1-47 for 48 h, and glutamine uptake was subsequently assessed. **C** Cells were transduced with the indicated USP14 and/or SLC1A5 expression or knockdown constructs, and glutamine uptake was measured. **D** Intracellular glutathione (GSH) levels were measured in cells transduced with shNC, shUSP14-1, or shUSP14-2. **E** Intracellular GSH levels were measured in cells treated with IU1-47 for 48 h. **F** Intracellular GSH levels were measured in cells transduced with the indicated USP14 and/or SLC1A5 constructs. **G** Intracellular reactive oxygen species (ROS) levels were determined in cells transduced with shNC, shUSP14-1, or shUSP14-2. **H** Intracellular ROS levels were measured in cells treated with IU1-47 for 48 h. **I** Intracellular ROS levels were determined in cells transduced with the indicated USP14 and/or SLC1A5 constructs. **J** Western blot analysis of SLC1A5, p-mTOR, mTOR, p-4EBP1, and 4EBP1 protein expression in cells transduced with shNC, shUSP14-1, or shUSP14-2. **K** Western blot analysis of SLC1A5, p-mTOR, mTOR, p-4EBP1, and 4EBP1 protein expression in cells treated with IU1-47. **L** Western blot analysis of SLC1A5, p-mTOR, mTOR, p-4EBP1, and 4EBP1 protein expression in cells transduced with the indicated USP14 and/or SLC1A5 constructs.Quantification of protein levels shown in (**J**–**L**) is provided in Supplementary Fig. S6A–C. Data are expressed as mean ± SEM (*p < 0.05, **p < 0.01, *n* = 3).

Given the essential role of SLC1A5 in driving glutamine metabolism, we then explored whether USP14 regulates glutamine metabolism-related pathways through SLC1A5. Knockdown of USP14 or treatment with USP14 inhibitor significantly reduced glutamine uptake in OC cells (Fig. 6A, B). Conversely, USP14 overexpression increased glutamine uptake, but this effect was eliminated by SLC1A5 knockdown. Importantly, re-expression of SLC1A5 restored the increase (Fig. 6C). Inhibition of USP14 via siRNA knockdown or IU1-47 treatment significantly lowered GSH production (Fig. 6D, E) and led to enhanced ROS accumulation (Fig. 6G, H), indicating increased oxidative stress. In contrast, USP14 overexpression produced the opposite effects, resulting in elevated GSH levels and reduced ROS accumulation. Notably, these protective effects conferred by USP14 overexpression were abolished by SLC1A5 knockdown and reinstated by SLC1A5 reintroduction (Fig. 6F, I). Furthermore, USP14 downregulation suppressed the expression of p-mTOR and p-4EBP1, indicating inactivation of the mTORC1 signaling pathway (Fig. 6J, K). Conversely, USP14 overexpression resulted in mTORC1 signaling pathway activation, an effect that was blocked by SLC1A5 silencing and rescued by SLC1A5 reintroduction (Fig. 6L). Together, these findings indicate that USP14, by stabilizing SLC1A5, indirectly regulates glutamine uptake and downstream metabolic signaling.

### USP14 enhances resistance to olaparib by modulating SLC1A5

**Fig. 7:**
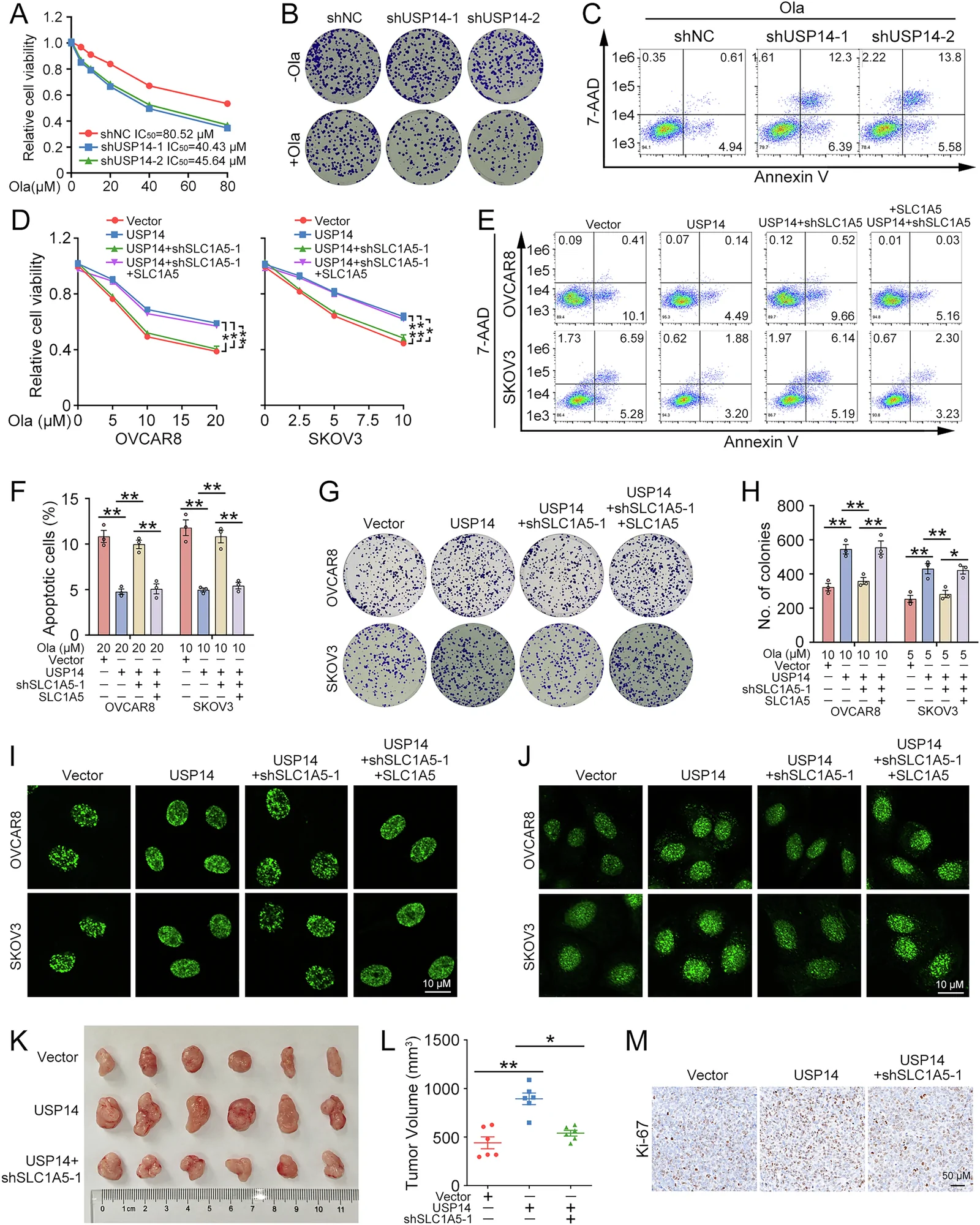
USP14 promotes olaparib resistance by interacting with SLC1A5. **A** SK/Ola cells were stably transduced with pLKO.1 control vector (shNC) or two independent USP14 shRNAs (shUSP14-1 and shUSP14-2). Cell viability was assessed by MTT assay following treatment with the indicated concentrations of olaparib for 72 h. **B** Colony formation assay was performed in SK/Ola cells treated with 20 μM olaparib. Quantification of colony numbers is provided in Supplementary Fig. S7E. **C** Representative flow cytometry plots showing apoptosis in SK/Ola cells treated with 40 μM olaparib for 72 h. Quantification of apoptotic cells is provided in Supplementary Fig. S7F. **D** OVCAR8 and SKOV3 cells were stably transduced with the indicated USP14 and/or SLC1A5 expression or knockdown constructs. Cell viability was evaluated by MTT assay following olaparib treatment for 72 h. **E** Representative flow cytometry plots showing apoptosis in OVCAR8 and SKOV3 cells treated with olaparib (OVCAR8: 20 μM; SKOV3: 10 μM) for 72 h. **F** Quantification of apoptotic cells shown in (**E**). **G** Colony formation assays were performed in OVCAR8 and SKOV3 cells treated with olaparib (OVCAR8: 10 μM; SKOV3: 5 μM). **H** Quantification of colony numbers shown in (**G**). **I** Representative immunofluorescence images of γH2AX foci in OVCAR8 and SKOV3 cells treated with olaparib (OVCAR8: 20 μM; SKOV3: 10 μM) for 48 h. Scale bar: 10 μm. Quantification of γH2AX foci is provided in Supplementary Fig. S7O. **J** Representative immunofluorescence images of RAD51 foci in OVCAR8 and SKOV3 cells treated with olaparib (OVCAR8: 20 μM; SKOV3: 10 μM) for 48 h. Scale bar: 10 μm. Quantification of RAD51 foci is provided in Supplementary Fig. S7P. **K** OVCAR8 cells (5 × 10^6^) stably expressing pCMV (Vector), pCMV-USP14 (USP14), and/or SLC1A5 shRNA 1 (shSLC1A5-1) were subcutaneously injected into nude mice (*n* = 6 per group). When tumors reached approximately 50 mm^3^ in volume, mice received intraperitoneal injections of olaparib (50 mg/kg) every other day. After 3 weeks of injection, tumors were harvested for analysis. Representative images of excised tumors are shown. **L** Quantification of tumor volumes at the experimental endpoint. **M** Representative IHC staining images of Ki-67 in xenograft tumors. Quantification of Ki-67-positive cells is provided in Supplementary Fig. S7Q. Data are expressed as the mean ± SEM (*p < 0.05, **p < 0.01, *n* = 3).

We first examined whether USP14 is involved in regulating PARPi resistance in OC. Western blot analysis showed higher USP14 protein levels in SK/Ola cells than in their parental counterparts (Supplementary Fig. S7A, B). To assess the functional role of USP14, we generated stable knockdown models in SK/Ola cells, with knockdown efficiency confirmed by western blot (Supplementary Fig. S7C, D). Functional assays revealed that USP14 knockdown significantly sensitized SK/Ola cells to PARPi. Specifically, MTT assays demonstrated that USP14-deficient cells exhibited enhanced drug sensitivity compared with control cells, as reflected by reduced cell viability (Fig. 7A). Colony formation assays further showed that the clonogenic capacity of USP14‑knockdown cells was substantially impaired under olaparib treatment (Fig. 7B). Moreover, flow cytometric analysis revealed that USP14 deficiency led to increased apoptosis following olaparib exposure (Fig. 7C). To further validate the role of USP14 in PARPi resistance, we also established USP14 knockdown in OVCAR8 and SKOV3 cells (Supplementary Fig. S7G, H). MTT assays confirmed that USP14 knockdown enhanced olaparib sensitivity in these cell lines (Supplementary Fig. S7I, J). Apoptosis analysis verified that USP14 knockdown promoted cell death (Supplementary Fig. S7K, L), and colony formation assays showed reduced clonogenic survival upon USP14 inhibition (Supplementary Fig. S7M, N). Together, these results demonstrate that USP14 acts as a key mediator of PARPi resistance in OC.

We next examined whether USP14-mediated olaparib resistance depends on SLC1A5. MTT assays showed that SLC1A5 knockdown abolished the resistance conferred by USP14 overexpression, and this effect was reversed upon SLC1A5 re-expression (Fig. 7D). Flow cytometry analysis demonstrated that SLC1A5 knockdown attenuated USP14-driven olaparib resistance, as evidenced by increased apoptosis, whereas reintroduction of SLC1A5 restored resistance by reducing apoptosis (Fig. 7E, F). Consistently, colony formation assays showed that SLC1A5 depletion impaired the clonogenic survival of USP14-overexpressing cells under olaparib treatment, an effect that was reversed upon SLC1A5 re-expression (Fig. 7G, H). IF staining revealed that USP14 overexpression reduced γH2AX foci formation (Fig. 7I) while increasing RAD51 foci accumulation (Fig. 7J) upon olaparib treatment. These alterations were abolished by SLC1A5 knockdown and restored by SLC1A5 re-expression (Fig. 7I, J). We next evaluated whether USP14 promotes olaparib resistance through SLC1A5 in a xenograft mouse model. USP14 overexpression increased olaparib resistance, whereas SLC1A5 knockdown abrogated this effect (Fig. 7K, L). IHC staining for Ki-67 further supported the functional interplay between USP14 and SLC1A5 in regulating tumor growth (Fig. 7M). Together, these results suggest that USP14 and SLC1A5 cooperatively drive olaparib resistance in OC.

### SLC1A5 inhibitor synergizes with olaparib against OC in vivo

**Fig. 8:**
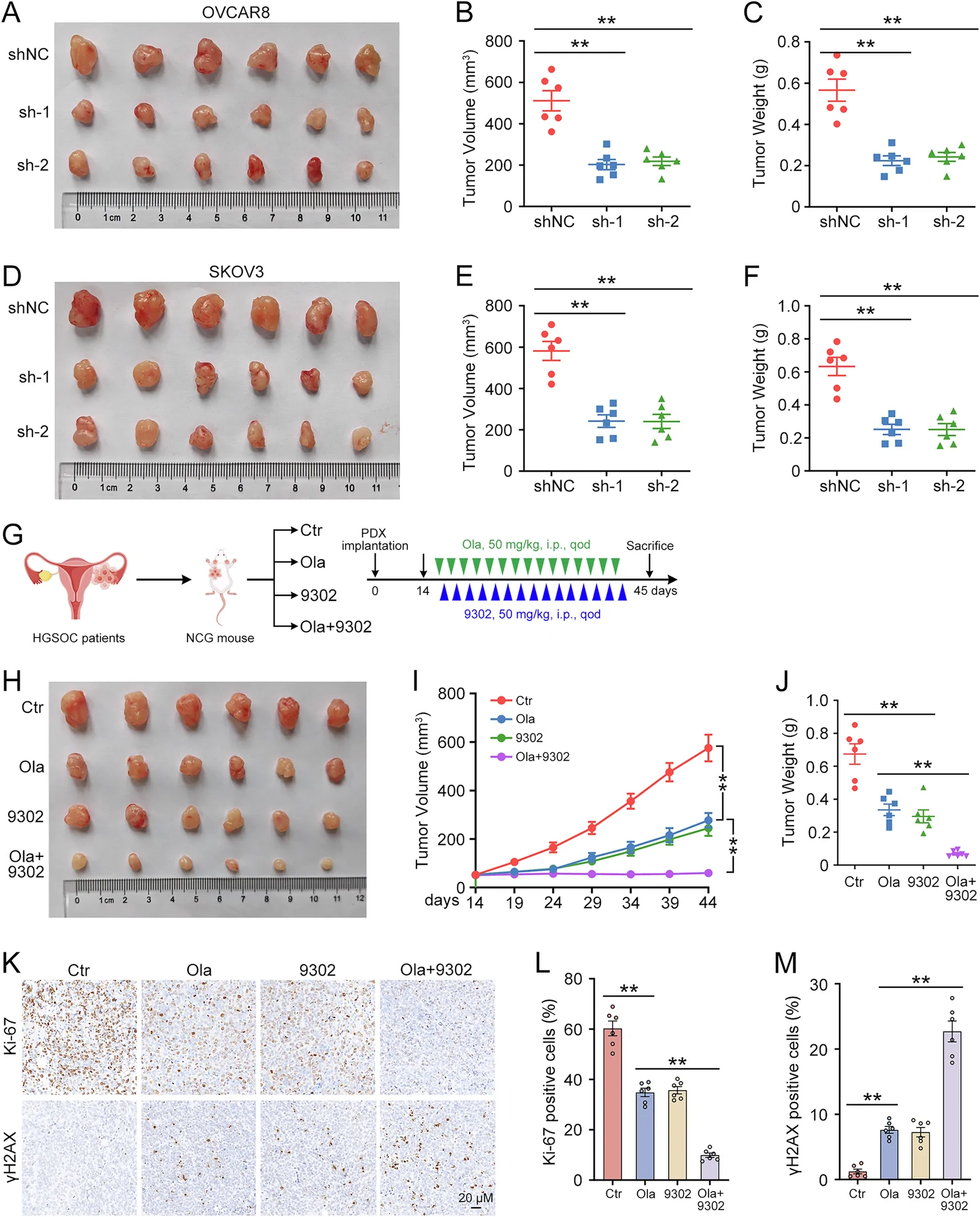
SLC1A5 inhibitor V-9302 enhances the efficacy of olaparib in vivo. **A** OVCAR8 cells (5 × 10^6^) stably expressing pLKO.1 (shNC), SLC1A5 shRNA 1 (sh-1), or SLC1A5 shRNA 2 (sh-2) were subcutaneously injected into nude mice (*n* = 6 per group). When tumors reached approximately 50 mm^3^ in volume, mice received intraperitoneal injections of olaparib (50 mg/kg) every other day. Tumors were harvested after 3 weeks of treatment. Representative images of excised tumors are shown. **B** Quantification of tumor volumes at the experimental endpoint. **C** Final tumor weights at the experimental endpoint. **D** SKOV3 cells (5 × 10^6^) stably expressing pLKO.1 (shNC), SLC1A5 shRNA 1 (sh-1), or SLC1A5 shRNA 2 (sh-2) were subcutaneously injected into nude mice (*n* = 6 per group). When tumors reached approximately 50 mm^3^ in volume, mice received intraperitoneal injections of olaparib (50 mg/kg) every other day. Tumors were harvested after 3 weeks of treatment. Representative tumor photographs are shown. **E** Quantification of tumor volumes at the experimental endpoint. **F** Final tumor weights at the experimental endpoint. **G** For the PDX model, tumor tissues from established PDX tumors were passaged into recipient NCG mice. Two weeks after implantation, mice were randomly assigned to four groups: vehicle control (Ctr), olaparib (Ola), V-9302 (9302), and olaparib combined with V-9302 (Ola+9302). Mice received intraperitoneal injections of olaparib (50 mg/kg), V-9302 (50 mg/kg), or the combination every other day. Following 4 weeks of treatment, the mice were euthanized and subjected to further analysis. **H** Representative images of excised PDX tumors from each group. **I** Tumor growth curves of PDX tumors during the treatment period. **J** Final tumor weights at the experimental endpoint. **K** Representative IHC staining images of Ki-67 and γH2AX in PDX tumor tissues. Scale bar: 20 µm. **L** Quantification of Ki-67-positive staining shown in (**K**). **M** Quantification of γH2AX-positive staining shown in (**K**). Data are expressed as the mean ± SEM (*p < 0.05, **p < 0.01, *n* = 6).

We first evaluated the effect of SLC1A5 knockdown on PARPi resistance using xenograft mouse models. OVCAR8 cells (5 × 10^6^) with or without SLC1A5 knockdown were subcutaneously injected into nude mice (*n* = 6 per group). When tumors reached an average volume of 50 mm^3^, mice received intraperitoneal injections of olaparib (50 mg/kg) every other day. Tumors were harvested after three weeks of treatment. The results showed that tumors in the SLC1A5 knockdown group were significantly smaller and lighter than those in the control group (Fig. 8A–C). These findings were further validated in a xenograft model established with SKOV3 cells (Fig. 8D–F). To assess the synergistic effect of SLC1A5 inhibitor and olaparib in vivo, we established a PDX model of HGSOC by subcutaneously injecting fresh tumor homogenate into NCG mice. Mice were then treated every other day with olaparib (50 mg/kg), V-9302 (50 mg/kg), or their combination (Fig. 8G). Tumors were harvested after four weeks of treatment. Compared with the control group, intraperitoneal administration of either V-9302 or olaparib alone reduced tumor volumes, and the combination therapy resulted in more pronounced suppression of tumor growth (Fig. 8H–J). Furthermore, IHC analysis revealed that combined treatment was associated with lower Ki-67 expression and a higher proportion of γH2AX-positive cells (Fig. 8K–M). Collectively, these results suggest that combining an SLC1A5 inhibitor with olaparib represents a promising strategy to overcome PARPi resistance in OC.

## Discussion

PARPi have revolutionized the treatment landscape for ovarian cancer. However, resistance to PARPi therapy significantly limits their long-term efficacy, posing a major clinical challenge [30]. In this study, we uncover a novel metabolic axis, USP14–SLC1A5, that plays a critical role in driving PARPi resistance in OC. We demonstrated that SLC1A5-mediated glutamine metabolism was significantly upregulated in PARPi-resistant OC cells. Genetic or pharmacological inhibition of SLC1A5 sensitized OC cells to PARPi both in vitro and in PDX models. Besides, the DUB USP14 was found to interact with and stabilize SLC1A5 by removing its K48-linked ubiquitin chains. Our findings establish a mechanistic link between protein deubiquitination, glutamine metabolism, and therapeutic resistance, offering new insights into potential strategies to overcome PARPi resistance in ovarian cancer.

Metabolic reprogramming is increasingly recognized as a driver of cancer progression and treatment failure, and glutamine addiction represents one of the most prominent metabolic vulnerabilities in rapidly proliferating tumors [8]. Glutamine metabolism plays a crucial supportive role in replication stress and the DNA damage response by supplying nitrogen for nucleotide synthesis, sustaining TCA cycle anaplerosis for energy production, and generating GSH/NADPH to buffer oxidative stress, while simultaneously activating pro-survival signaling pathways such as mTORC1 [31, 32]. Consequently, reprogramming glutamine metabolism can enhance the DNA damage repair capacity of tumor cells and promote their resistance to DNA-damaging therapies, including radiotherapy and chemotherapy. For instance, glutamine-induced ATP production and GSH synthesis have been shown to promote gemcitabine resistance in pancreatic cancer cells [33]. Inhibition of glutamine metabolism reverses paclitaxel resistance in OC cells via the mTORC1 signaling pathway [34]. In this study, we demonstrated that USP14-stabilized SLC1A5 enhanced glutamine uptake, leading to increased cellular glutamine levels that activate the mTORC1 signaling pathway, boost GSH production, and promote ROS scavenging. Collectively, these mechanisms contributed to DNA damage repair and PARPi resistance in OC. Additionally, OC cells exhibited greater PARPi resistance when cultured in higher glutamine concentrations. Thus, targeting and modulating glutamine metabolism emerges as a promising therapeutic strategy to overcome PARPi resistance in OC.

Glutamine is transported across the plasma membrane by various members of the solute-linked carrier (SLC) families. Among these, SLC1A5 demonstrates high affinity and specificity for glutamine, positioning it as a key transporter. SLC1A5 expression is tightly regulated across multiple levels. Transcriptionally, it is upregulated by factors such as ATF4 or MYC and downregulated by RB [35–37]. At the post-translational level, SLC1A5 undergoes N-linked glycosylation, which modulates its membrane localization and functional activity [38]. The ubiquitination status of SLC1A5 also critically influences its protein stability and glutamine transport capacity. In breast cancer, SLC1A5 is subject to ubiquitination and degradation by the RNF5 in response to chemotherapy-induced endoplasmic reticulum (ER) stress [39]. Additionally, CRL3^SPOP^ has been identified as an E3 ubiquitin ligase targeting SLC1A5; its inactivation promotes glutamine uptake by inducing K48-linked polyubiquitination and subsequent SLC1A5 degradation [40]. However, the deubiquitinating enzyme (DUB) responsible for stabilizing the SLC1A5 protein remained unknown. In this study, we identified USP14 as the novel DUB that regulated SLC1A5 stability through direct interaction. Overexpression of USP14 decreased SLC1A5 ubiquitination and elevated its protein levels, while USP14 inhibition exhibited opposing effects. USP14 stabilized SLC1A5 by inhibiting its K48-linked polyubiquitination-mediated degradation. Additionally, the expression levels of USP14 and SLC1A5 exhibited a positive correlation in HGSOC samples. These findings enhance the understanding of the ubiquitination-dependent mechanisms regulating SLC1A5 protein expression.

SLC1A5 is frequently overexpressed in various tumor types, including ovarian cancer, positioning it as a potential biomarker and therapeutic target in oncology. The development of selective SLC1A5 inhibitors has shown encouraging antitumor activity. For example, the inhibitor GPNA has been reported to suppress cancer cell growth by reducing glutamine uptake, which subsequently decreases GSH production and increases ROS levels [41–43]. However, GPNA lacks specificity and also inhibits other Na⁺-dependent transporters. More selective agents such as V-9302 have since been developed. Pharmacological blockade of SLC1A5 with V-9302 attenuates cancer cell proliferation, promotes cell death, and elevates oxidative stress, contributing to antitumor responses both in vitro and in vivo [44]. Research on SLC1A5 inhibitors in ovarian cancer remains limited, with existing studies focusing mainly on V-9302. This compound has been shown to downregulate mTOR/S6K signaling and re-sensitize paclitaxel-resistant SKOV3 cells to paclitaxel [34]. In the present study, V-9302 synergized with olaparib in vitro, exacerbating DNA damage and apoptosis. The combination therapy also demonstrated superior tumor growth suppression in PDX models compared to either agent alone. Our findings provide a strong preclinical rationale for evaluating such combination strategies in ovarian cancer patients at risk of developing PARPi resistance.

While this study identifies the USP14–SLC1A5 axis as a key driver of PARPi resistance in ovarian cancer, it has several limitations. Although our findings were validated in vitro and in PDX models, additional clinical cohorts are needed to confirm the translational relevance of the USP14–SLC1A5 axis in predicting PARPi response. Moreover, the long-term safety and potential off-target effects of SLC1A5 inhibition, as well as upstream mechanisms driving USP14 activation under PARPi treatment, warrant further investigation.

In conclusion, our study elucidated a previously unrecognized mechanism of PARPi resistance in ovarian cancer, centered on the USP14-mediated stabilization of SLC1A5 and the subsequent reprogramming of glutamine metabolism. This stabilization enhanced glutamine uptake, leading to increased GSH synthesis, ROS scavenging, and activation of the mTORC1 signaling pathway. These metabolic and signaling adaptations collectively foster a cellular environment that facilitates DNA damage repair and reduces the cytotoxicity of olaparib. Importantly, combined treatment with SLC1A5 inhibitor and olaparib synergistically suppresses tumor growth both in vitro and in PDX models. Our findings advance the understanding of PARPi resistance mechanisms in OC and provide an experimental basis for developing SLC1A5 inhibitors as a strategy to overcome such resistance.

## Supplementary information

original western blots

Supplementary information

Supplementary Table 1

## Data Availability

The datasets used and/or analyzed during the current study are available from the corresponding author on reasonable request.
