# Supplementary material for: Targeting SLC1A5-mediated glutamine metabolism overcomes PARP inhibitor resistance in ovarian cancer
Source: Cell Death Dis. 2026 Jul 22;17(1):890. doi: 10.1038/s41419-026-09123-z (PMC13677755; doi:10.1038/s41419-026-09123-z)

Figure 1

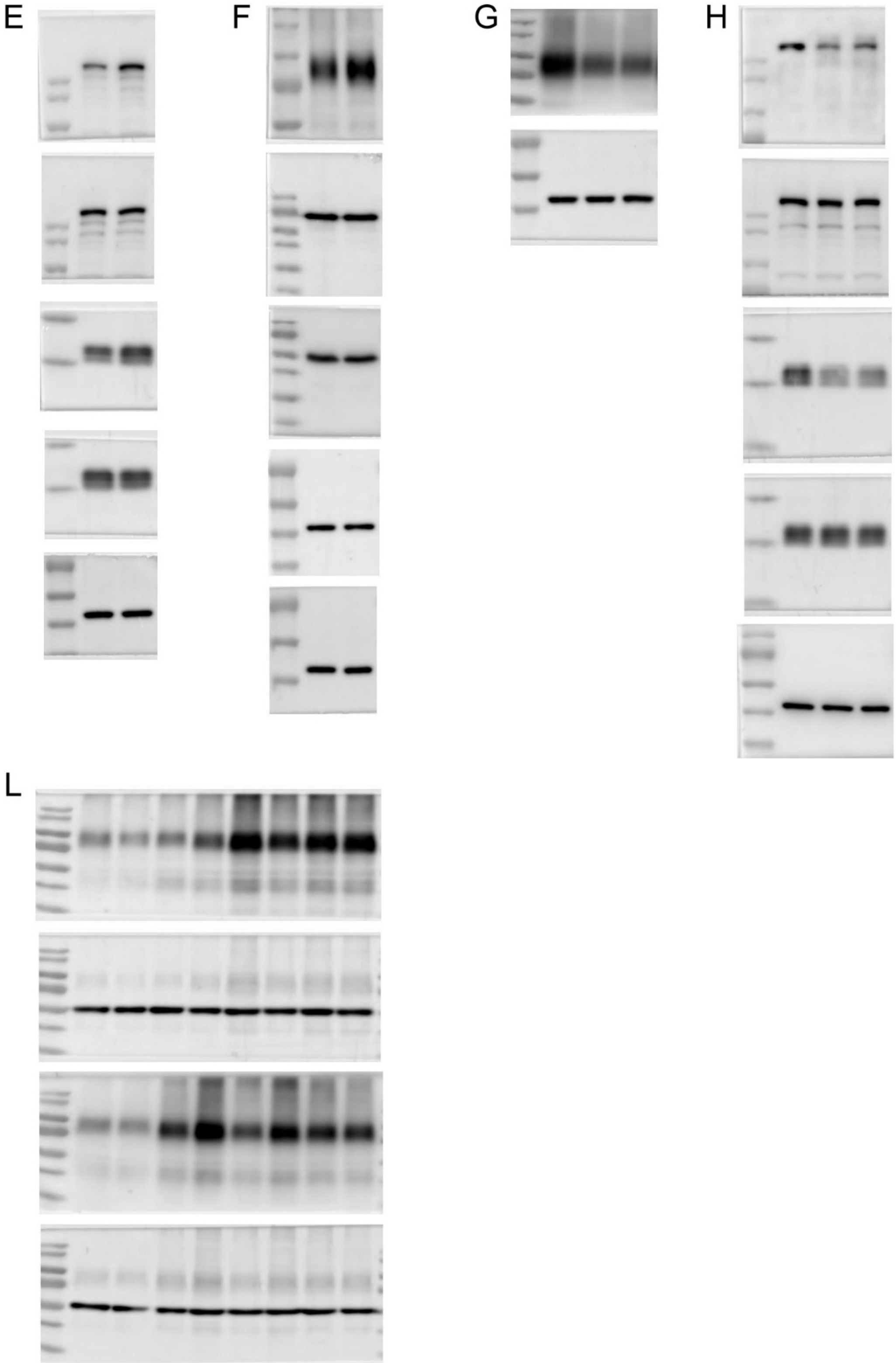

Figure 2

A

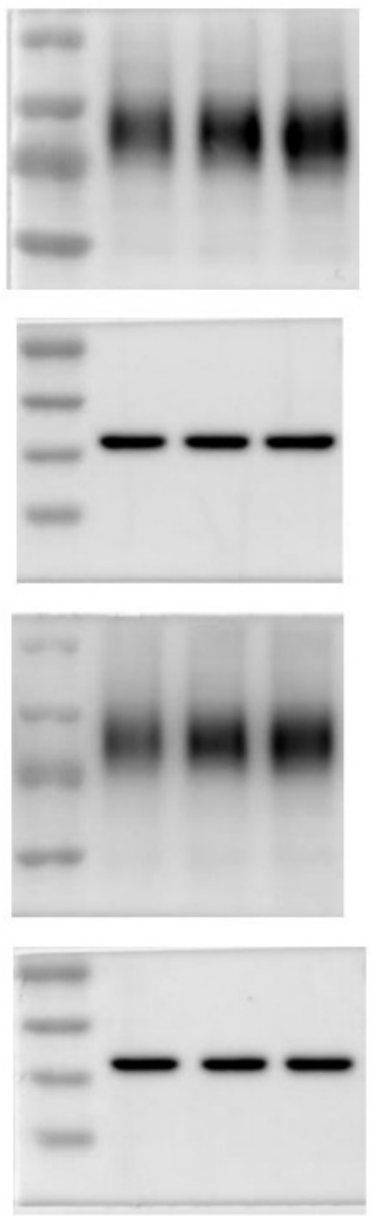

B

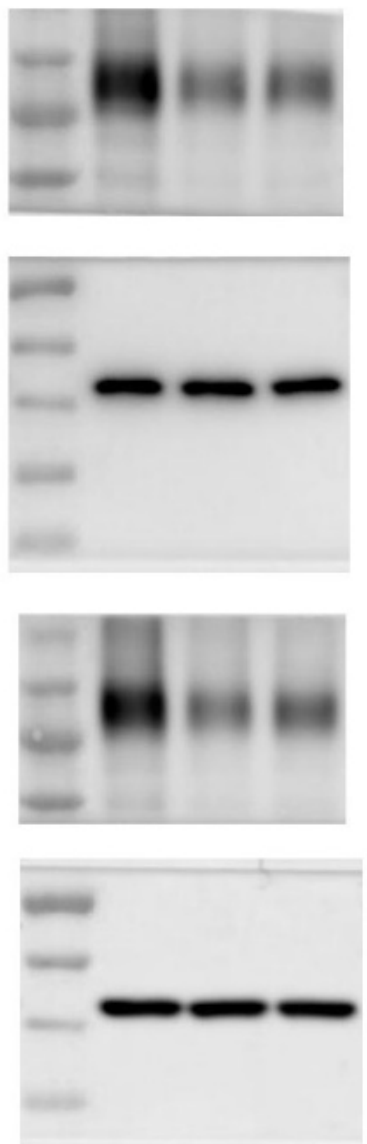

Figure 3

G

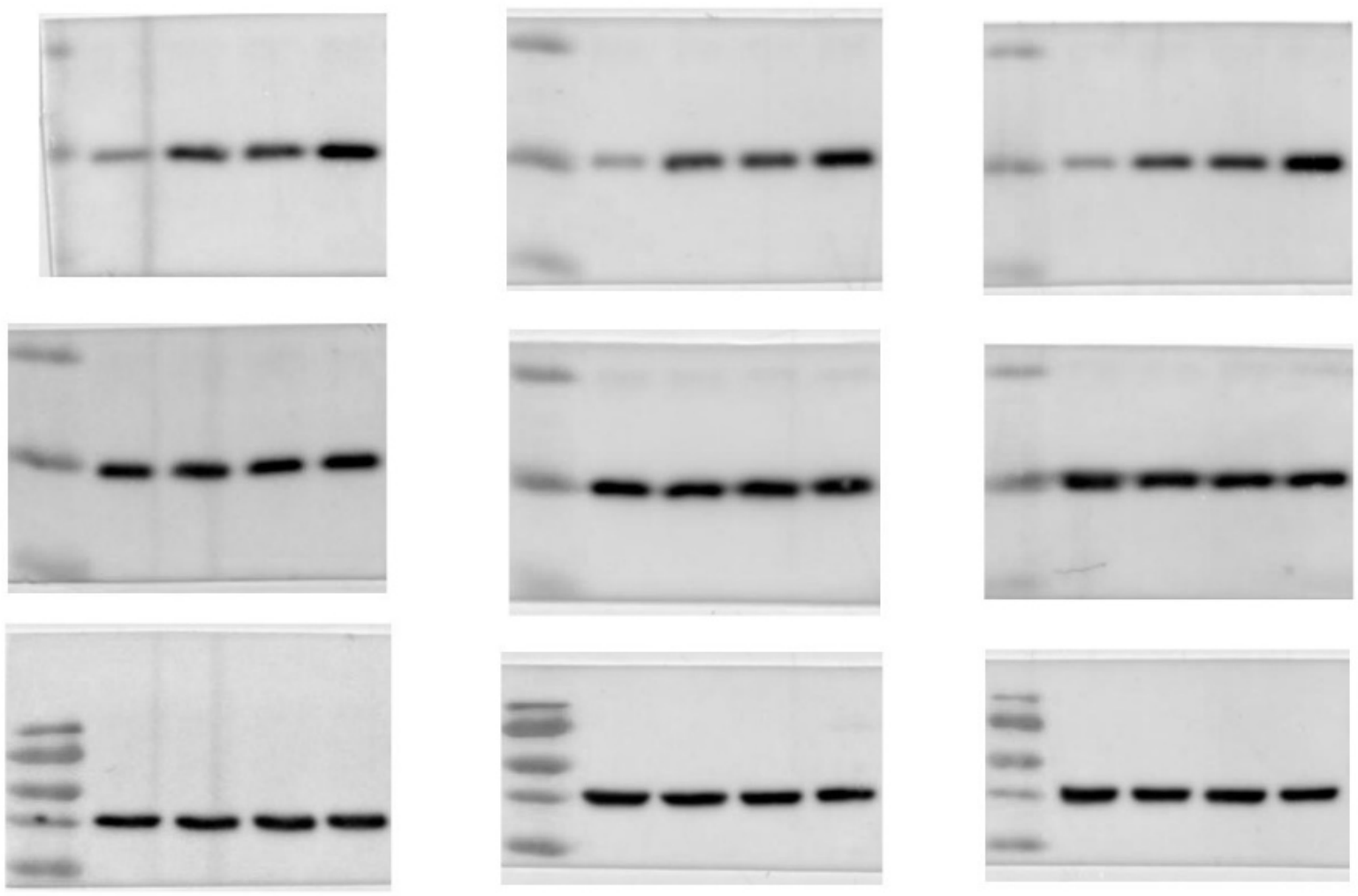

Figure 4

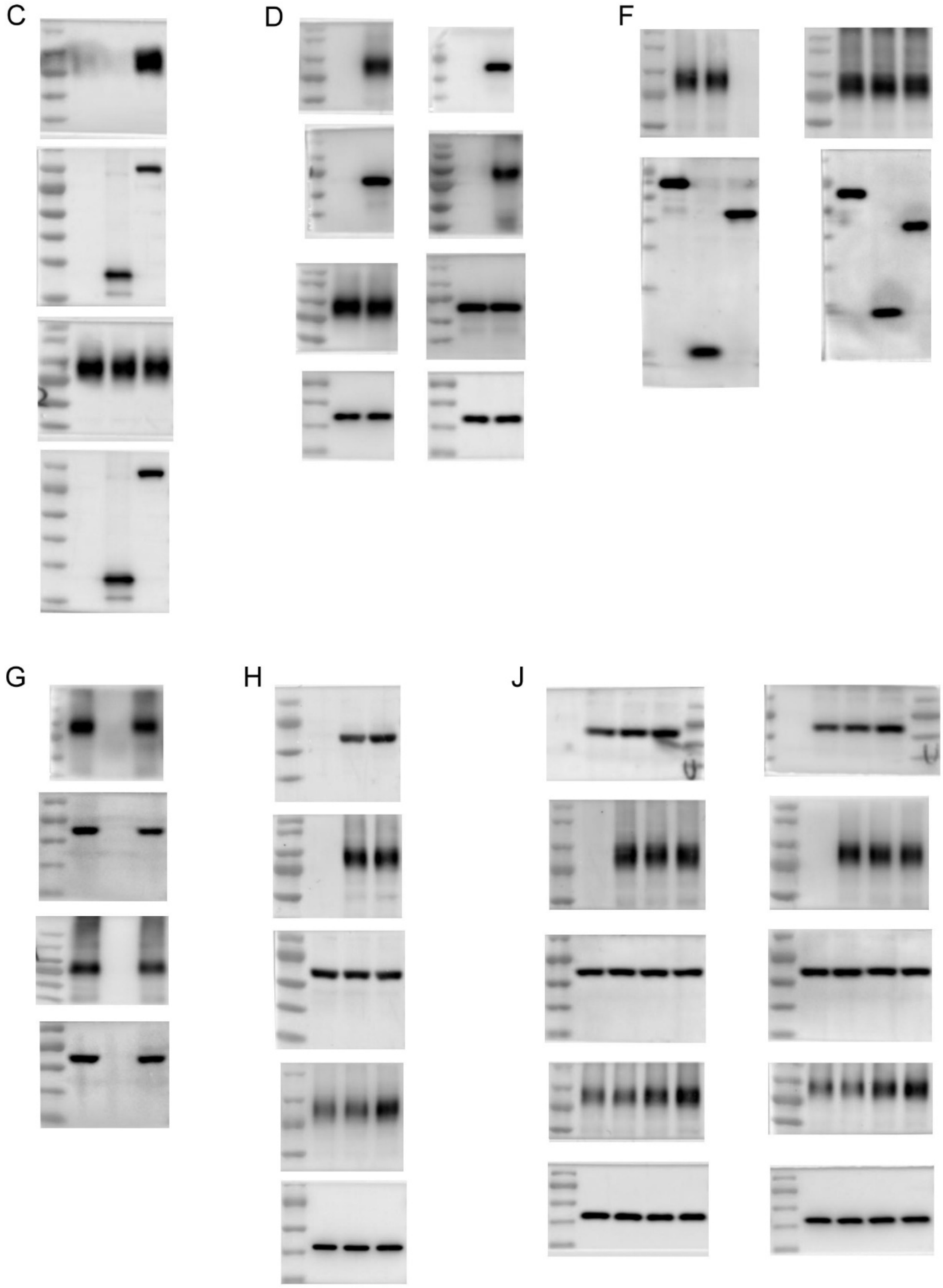

Figure 5

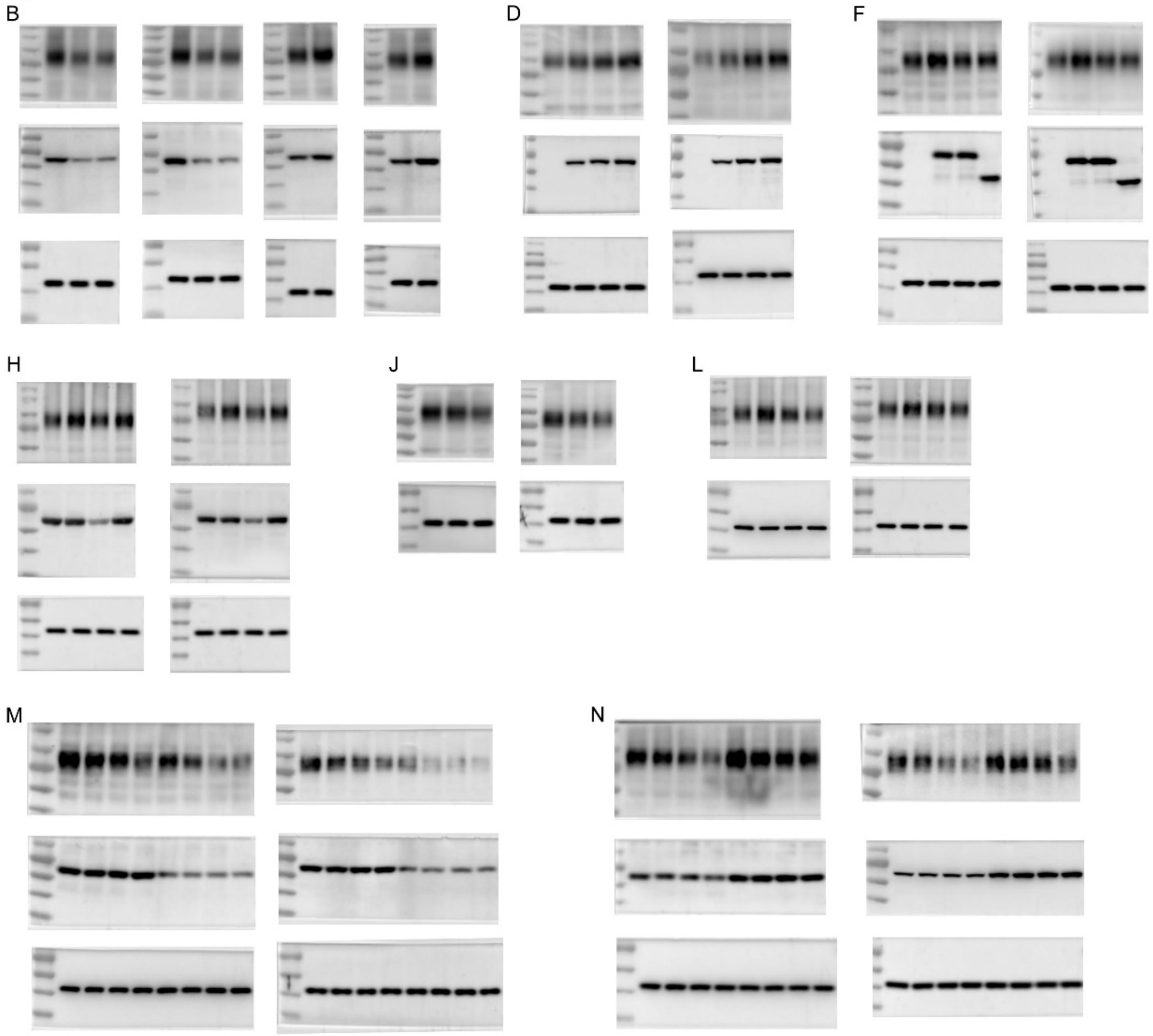

Figure 6

J K L

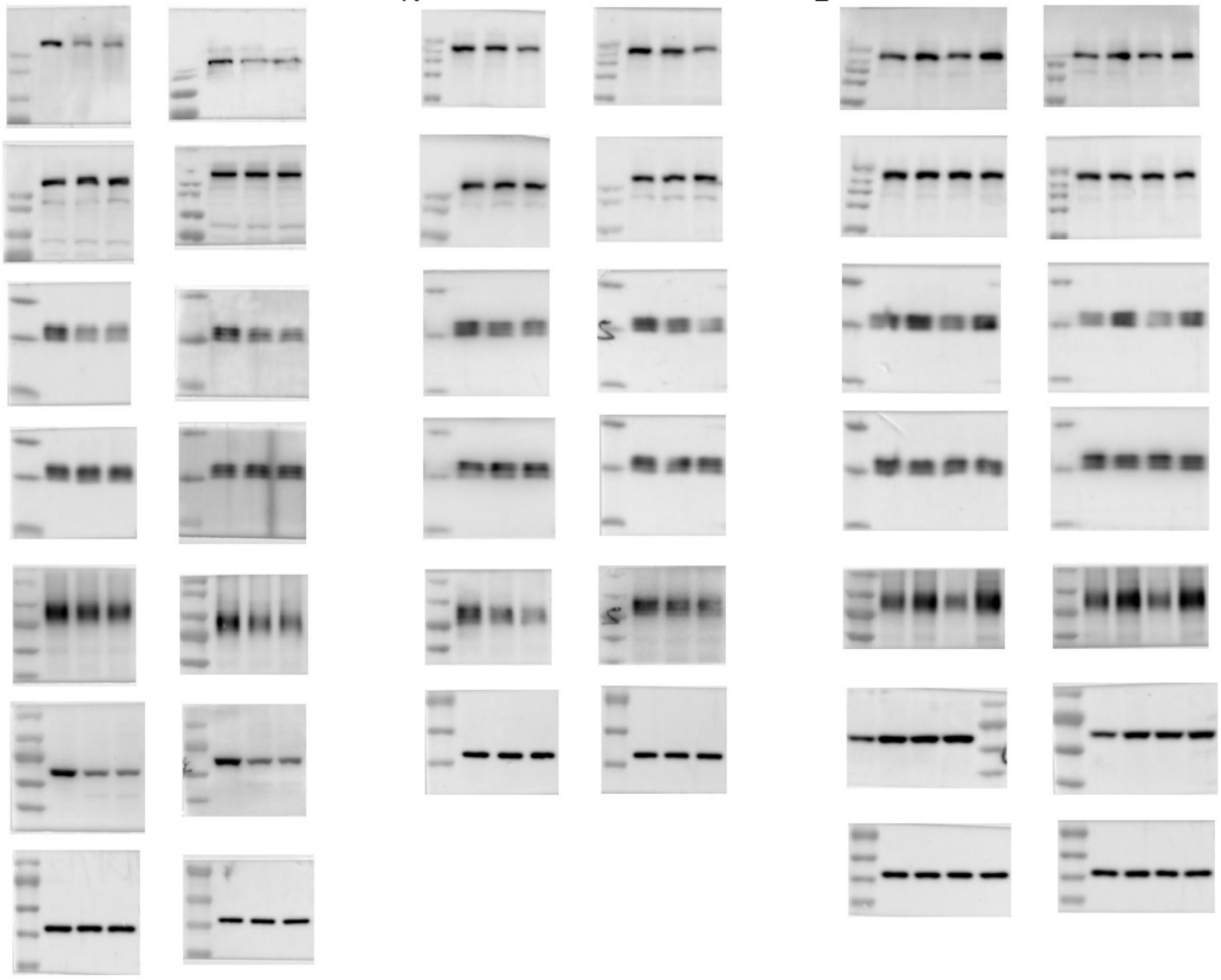

Figure S2

C

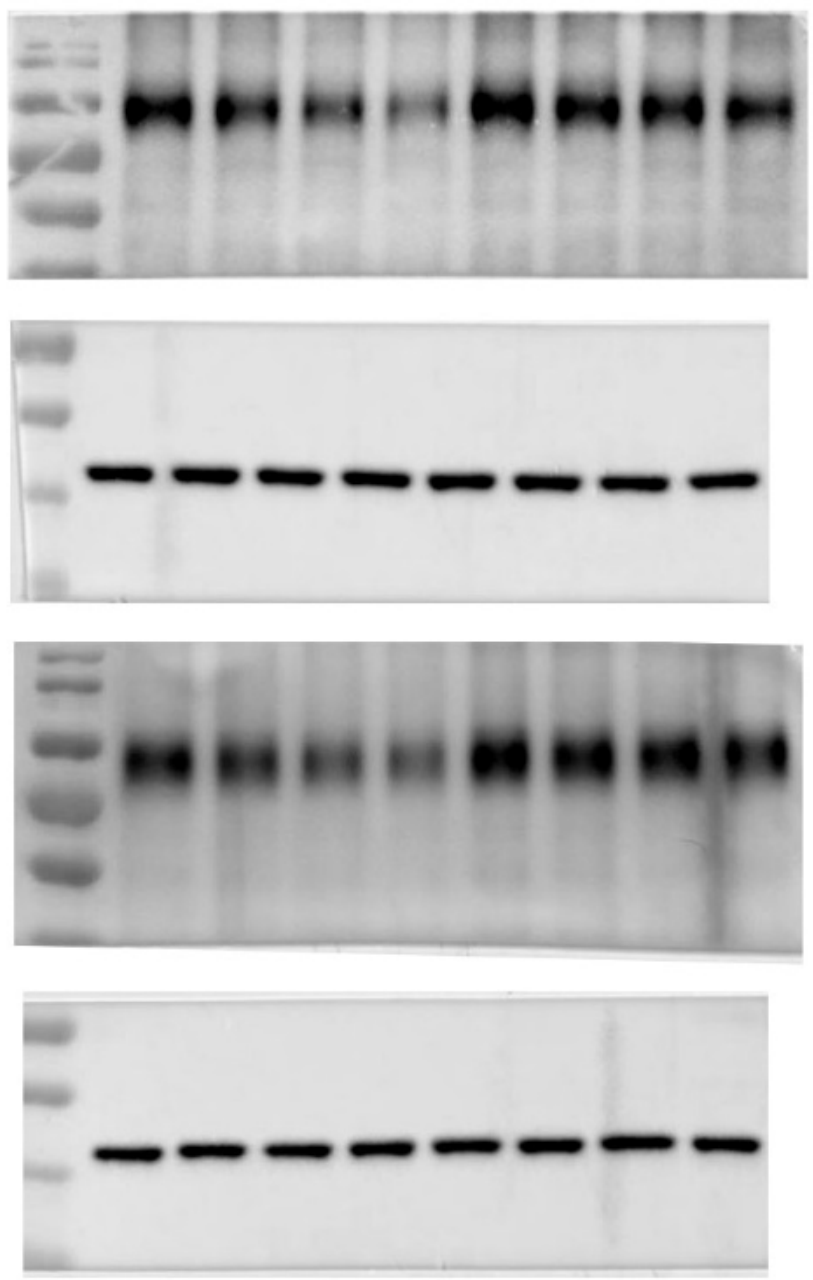

Figure S5

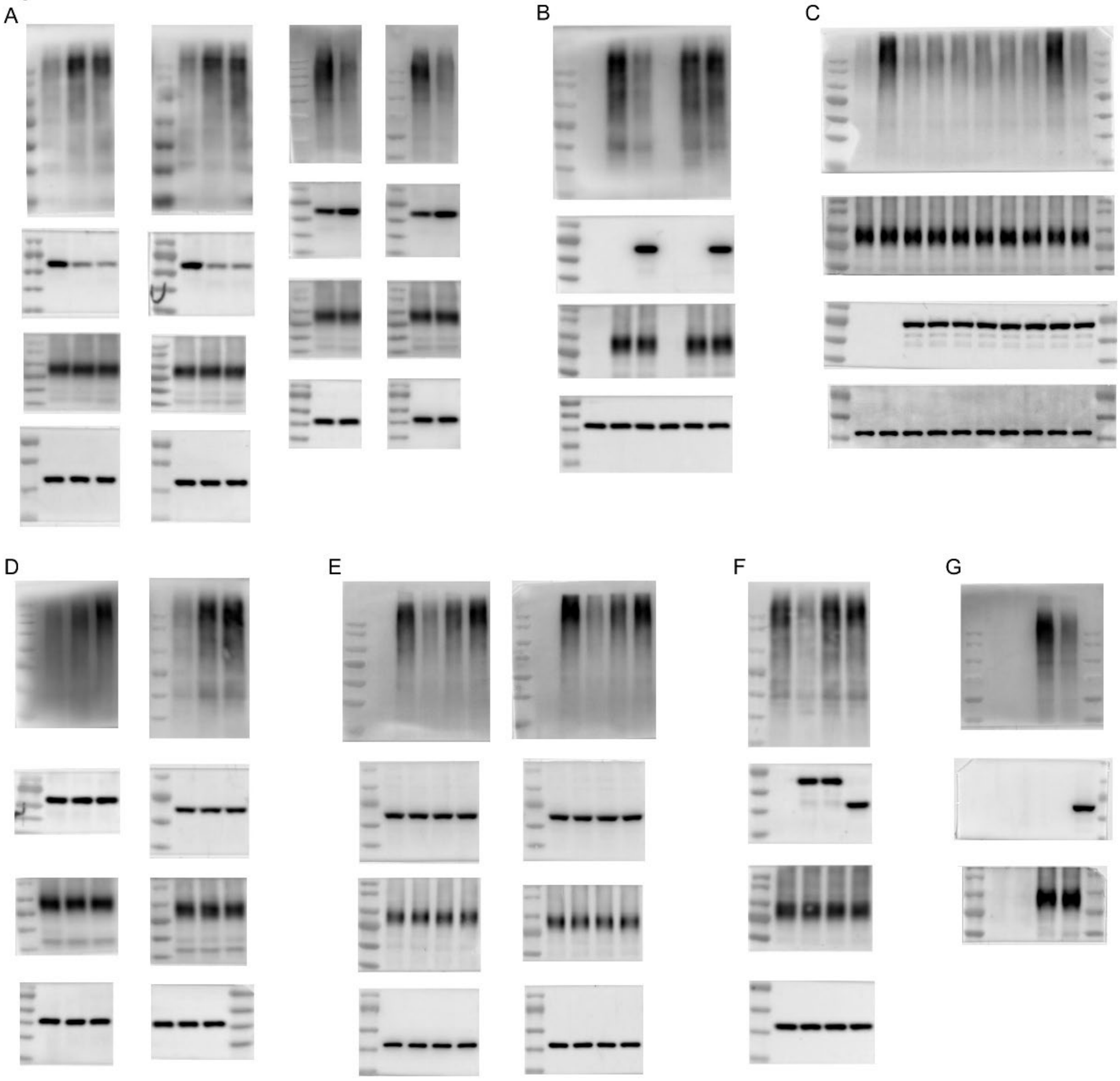

Figure S7

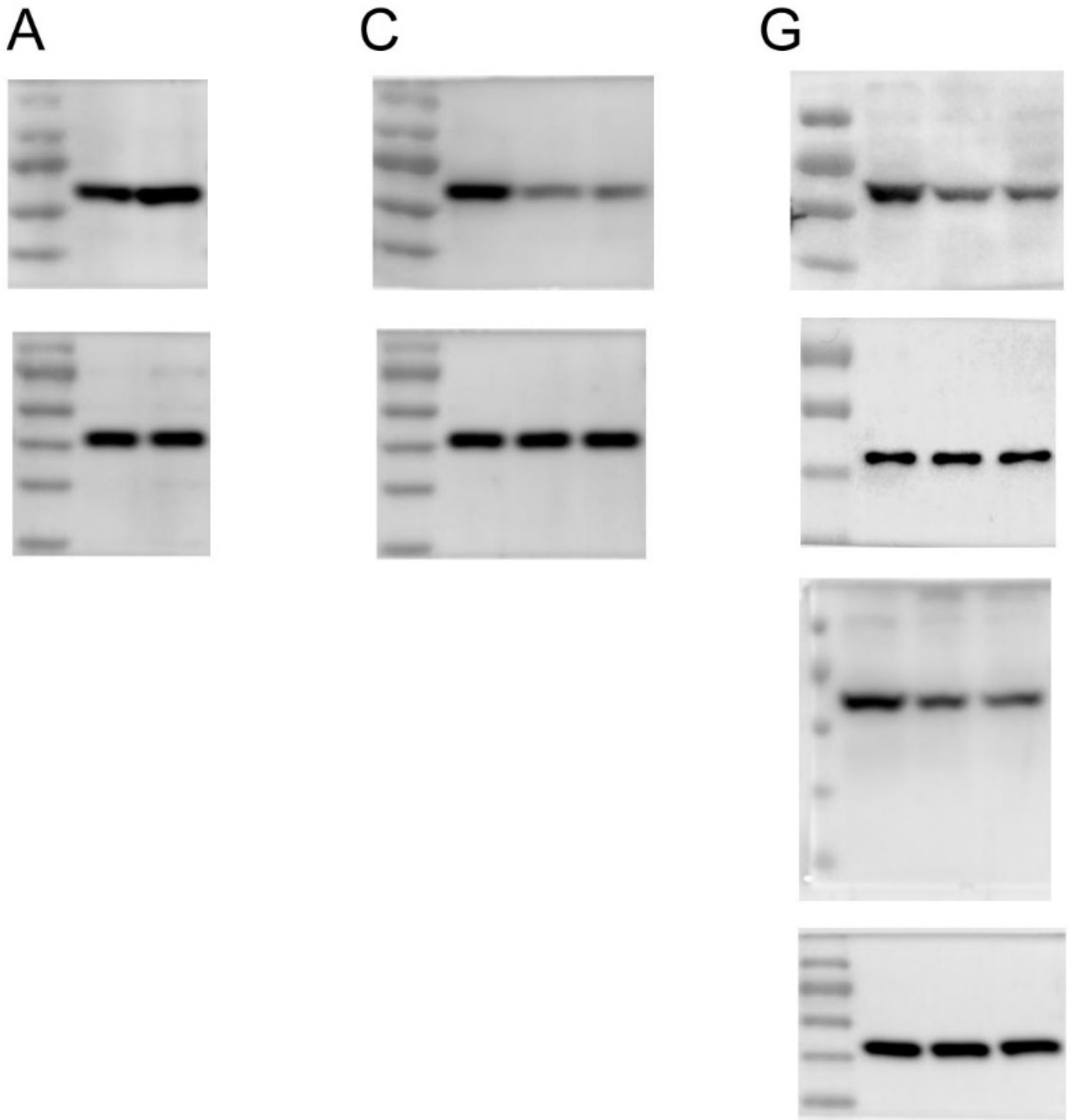

Supplement: Supplementary file 1 [file 41419_2026_9123_MOESM1_ESM.pdf]
