# Supplementary material for: Targeting SLC1A5-mediated glutamine metabolism overcomes PARP inhibitor resistance in ovarian cancer
Source: Cell Death Dis. 2026 Jul 22;17(1):890. doi: 10.1038/s41419-026-09123-z (PMC13677755; doi:10.1038/s41419-026-09123-z)
Supplement: Supplementary file 2 [file 41419_2026_9123_MOESM2_ESM.pdf]

Supplementary Figures

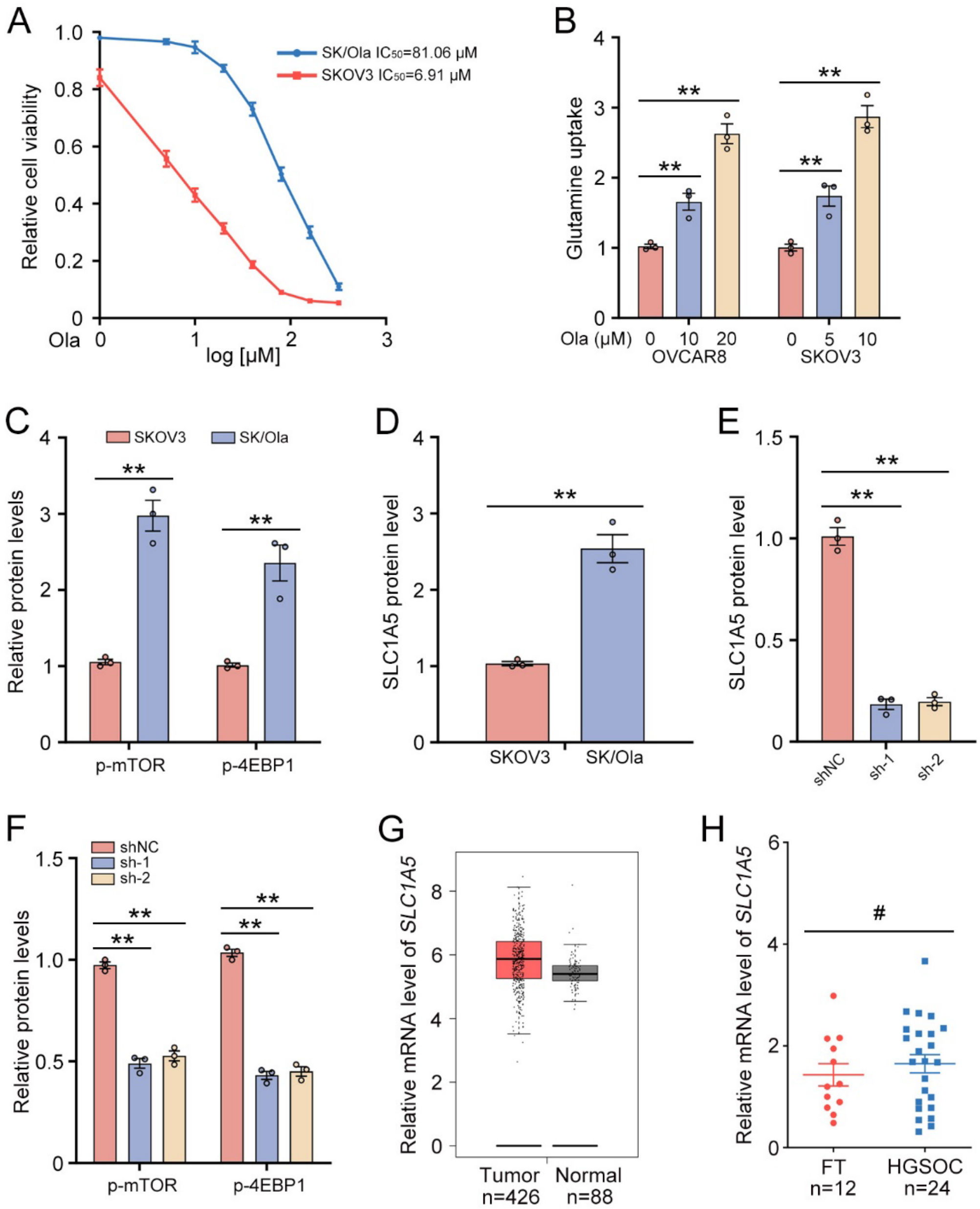

Figure S1. (A) SKOV3 and SK/Ola cells were treated with increasing concentrations of olaparib (Ola) for 72 h, and cell viability was assessed by MTT assay. (B) OVCAR8 and SKOV3 cells were treated with olaparib for 48 h, followed by assessment of glutamine uptake. (C–F) Quantification of protein levels from Figure 1E–H is presented. (G) Analysis of the TCGA dataset (http://gepia2.cancer-pku.cn) revealed the mRNA levels of *SLC1A5* in ovarian cancer tissues compared with normal controls. (H) qRT-PCR was performed to detect the mRNA levels of *SLC1A5* in normal fallopian tube (FT) tissues and high-grade serous ovarian cancer (HGSOC)

tissues (FT, n = 12; HGSOC, n = 24) from our cohort. Data are presented as the mean ± SEM ($^{\#}p > 0.05$, $^{**}p < 0.01$, n = 3).

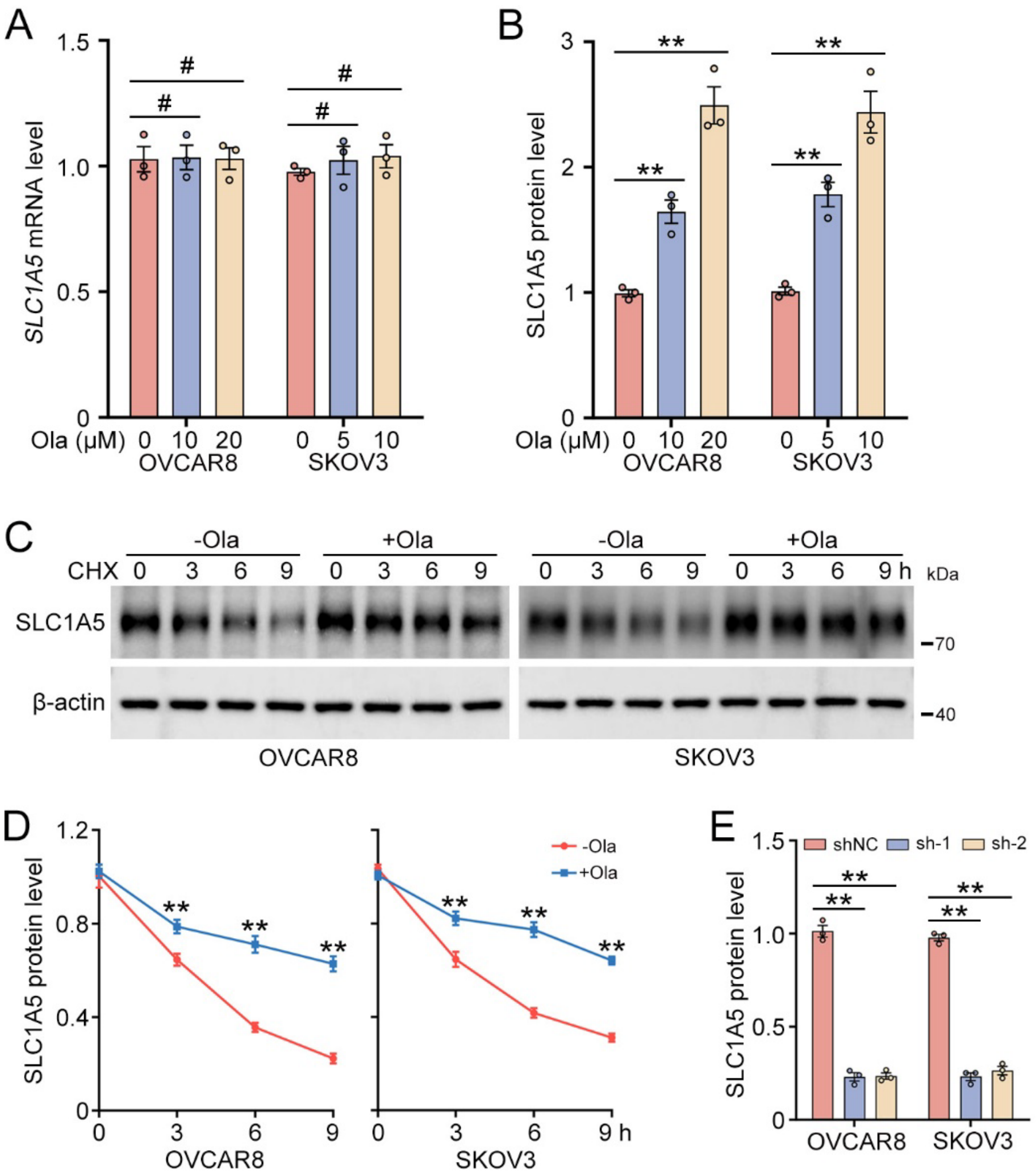

Figure S2. (A) qRT-PCR was performed to measure the mRNA levels of *SLC1A5* in cells treated with olaparib. (B) Quantification of protein levels from Figure 2A is presented. (C) Cells were pretreated with olaparib (OVCAR8: 20 μM; SKOV3: 10 μM) or control for 24 h, followed by incubation with CHX (100 μg/mL). Cell lysates were collected at 0, 3, 6, and 9 h after CHX addition, and SLC1A5 protein levels were analyzed by western blot. (D) Quantification of protein levels from (C) is presented. (E) Quantification of protein levels from Figure 2B is presented. Data are expressed as the mean ± SEM ($^{\#}p > 0.05$, $^{**}p < 0.01$, n = 3).

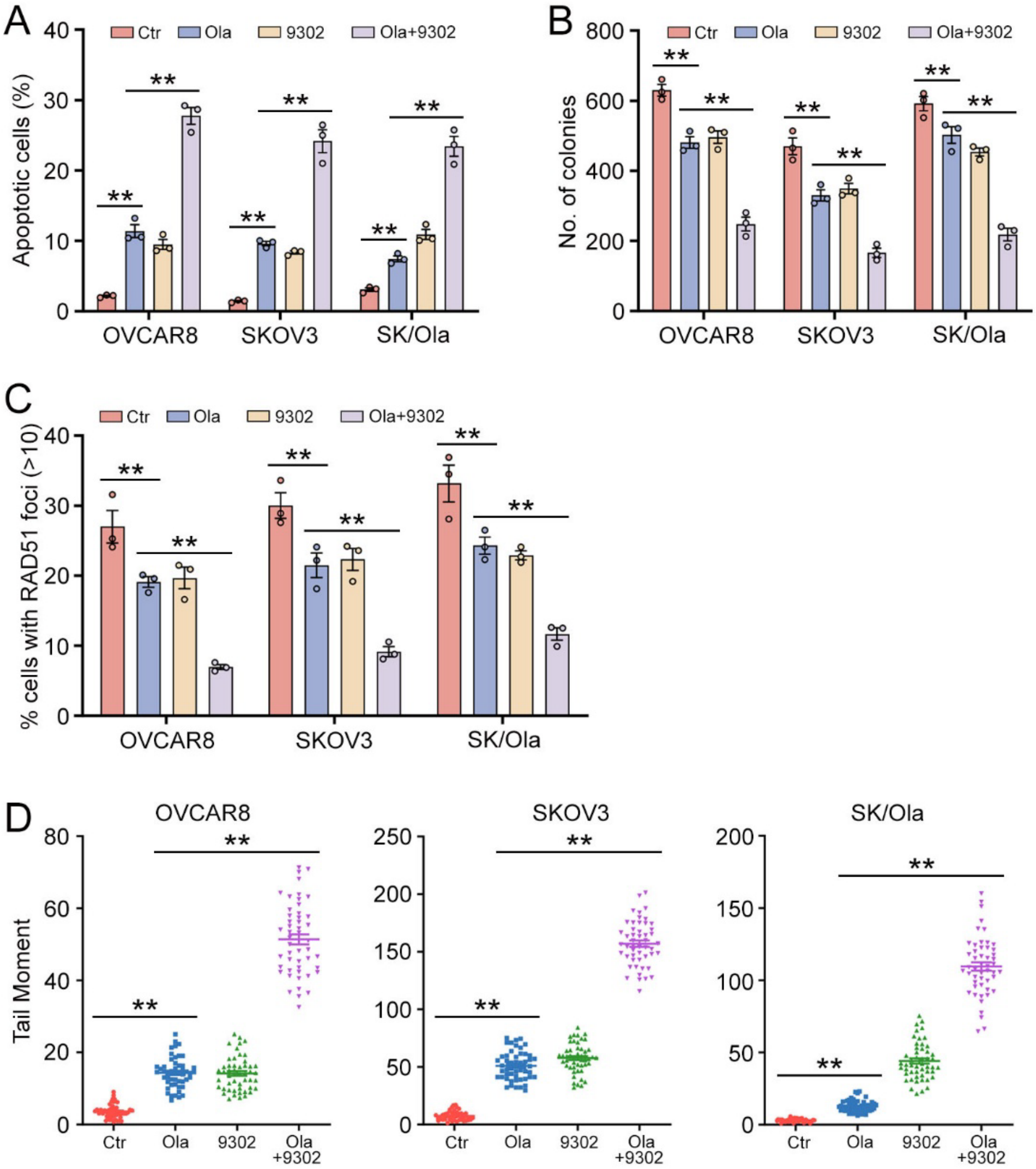

Figure S3. (A) The proportion of apoptotic cells from Figure 3C is quantified. (B) The number of colonies from Figure 3D is quantified. (C) Quantification of the percentage of cells with RAD51 foci (>10) in Figure 3E. (D) Quantification of the Tail moment of comet assay in Figure 3F. Data are expressed as the mean ± SEM (**p < 0.01, n = 3).

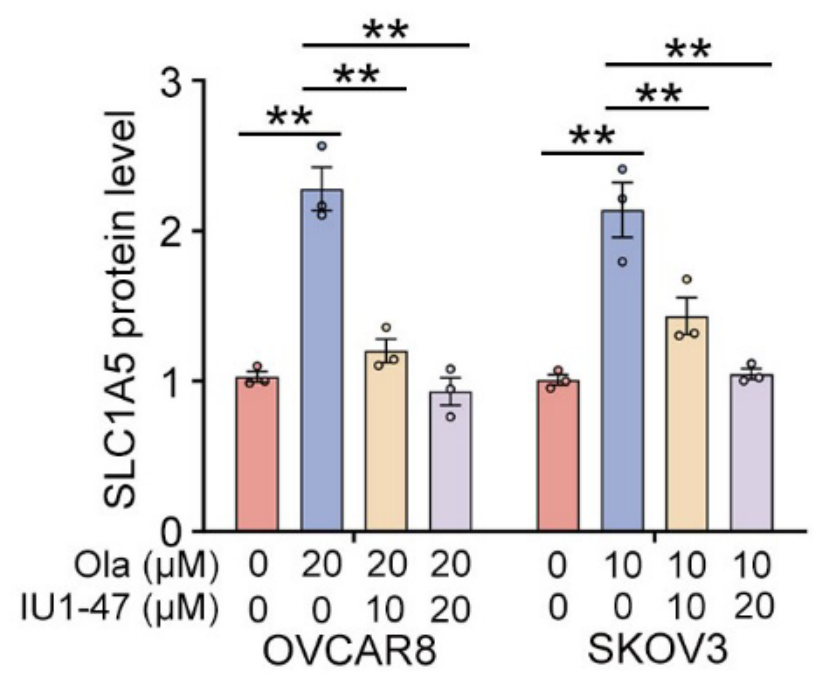

Figure S4. Quantification of the protein levels of SLC1A5 in Figure 5L. Data are expressed as the mean ± SEM (**$p < 0.01$, n = 3).

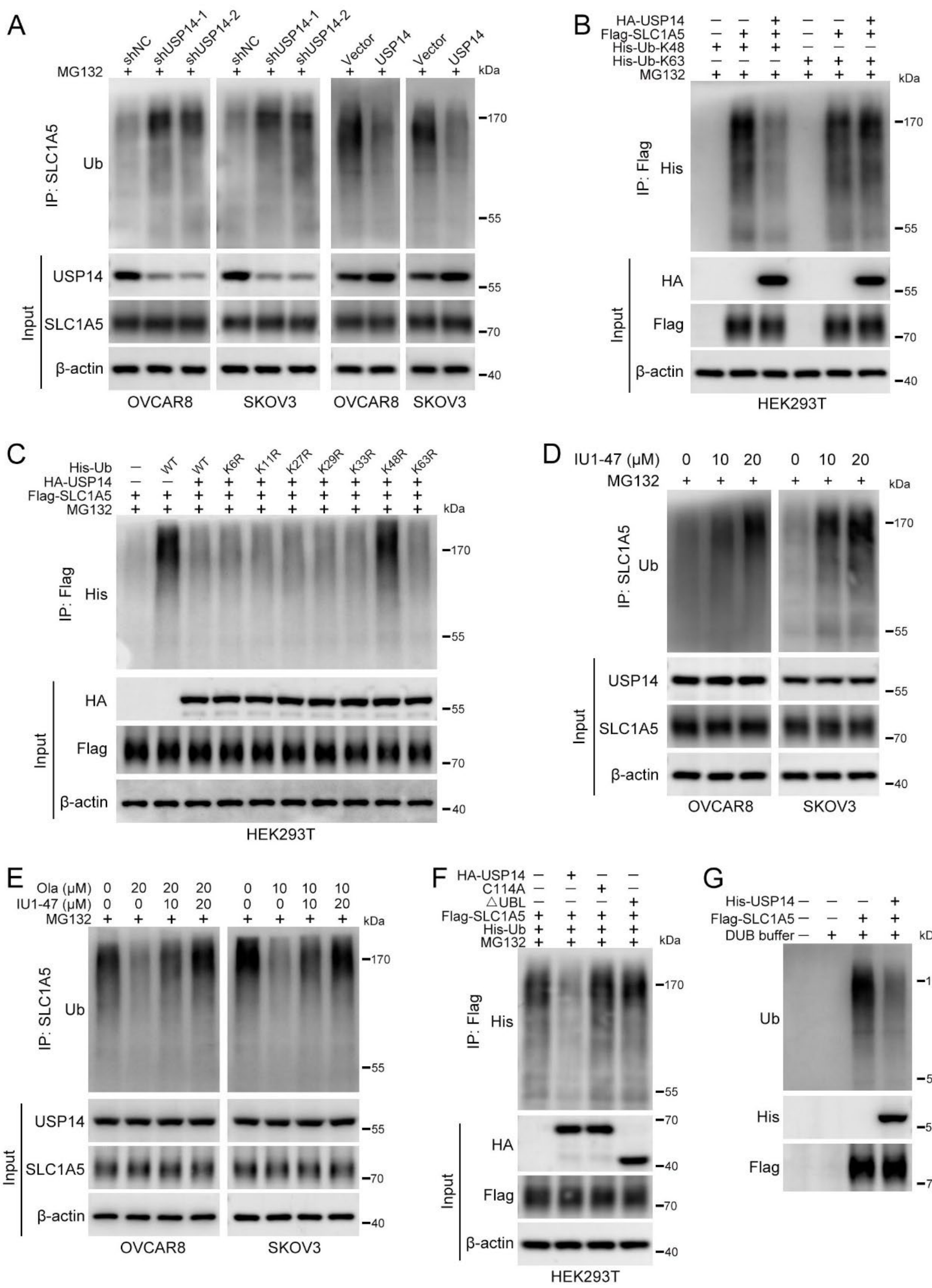

Figure S5. USP14 regulates the ubiquitination of SLC1A5. (A) A ubiquitination assay was conducted to evaluate SLC1A5 ubiquitination in cells with USP14 knockdown or

overexpression. Co-IP was performed using anti-SLC1A5 antibody, followed by immunoblotting with anti-Ub antibody. Cells were treated with MG132 (10 μM) for 6 h prior to sample collection. (B) HEK293T cells were transfected with HA-USP14, Flag-SLC1A5, His-Ub-K48, and His-Ub-K63 for 48 h, with MG132 (10 μM) added 6 h before harvest. Co-IP was performed using anti-Flag beads, and immunoblots were probed with the specified antibodies. (C) HEK293T cells were transfected with HA-USP14, Flag-SLC1A5, and His-Ub constructs (WT, K6R, K11R, K27R, K29R, K33R, K48R, or K63R) for 48 h, with MG132 (10 μM) added 6 h prior to harvest. Co-IP was conducted with anti-Flag beads, followed by immunoblotting with the indicated antibodies. (D) Cells were treated with IU1-47 for 48 h, with MG132 (10 μM) added 6 h before harvest. Co-IP was performed using an anti-SLC1A5 antibody, and immunoblots were probed with the specified antibodies. (E) Cells were treated with IU1-47 and/or olaparib for 48 h, with MG132 (10 μM) added 6 h prior to harvest. Co-IP was conducted with an anti-SLC1A5 antibody, followed by immunoblotting with the indicated antibodies. (F) HEK293T cells were transfected with HA-USP14, HA-USP14^{C114A} (C114A), HA-USP14$^{\Delta UBL}$ (ΔUBL), Flag-SLC1A5, and His-Ub for 48 h, with MG132 (10 μM) added 6 h before harvest. Co-IP was performed using anti-Flag beads, and immunoblots were probed with the specified antibodies. (G) An *in vitro* deubiquitination assay of USP14 was performed. Flag-SLC1A5 and HA-Ub were co-transfected into HEK293T cells for 48 h, followed by treatment with MG132 (10 μM) for 6 h. Flag-SLC1A5 (including its ubiquitinated form) was pulled down and incubated with purified His-USP14 for analysis.

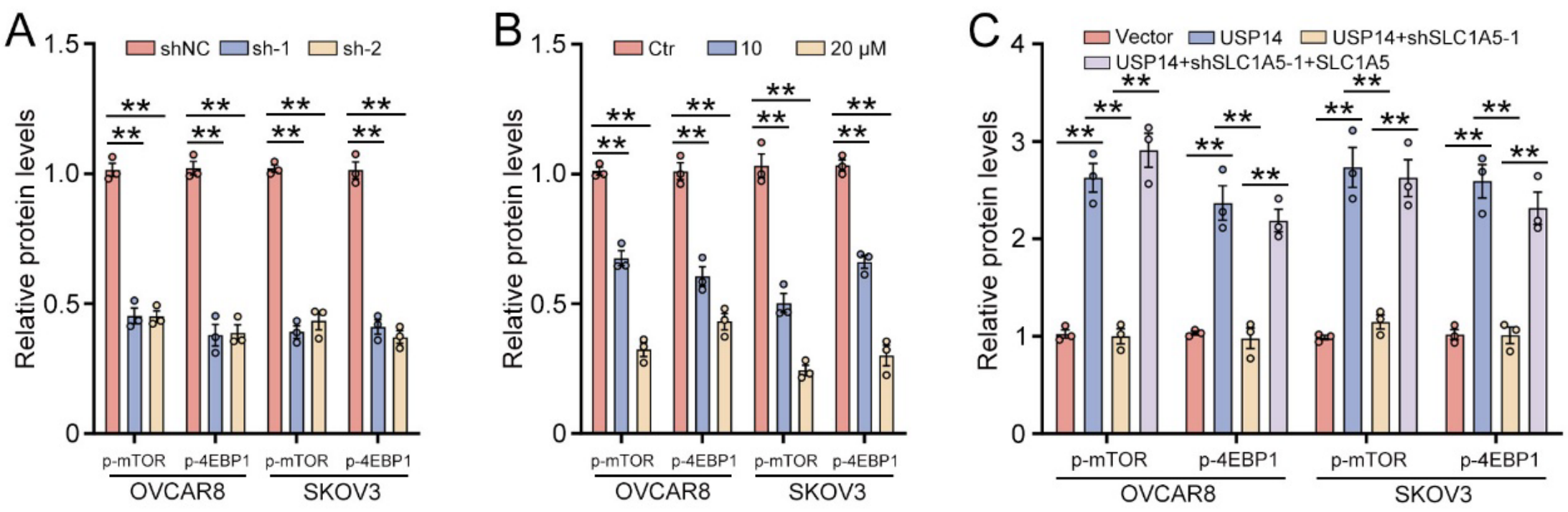

Figure S6. (A–C) Quantification of the protein levels in Figure 6J–L. Data are presented as the mean ± SEM (**p < 0.01, n = 3).

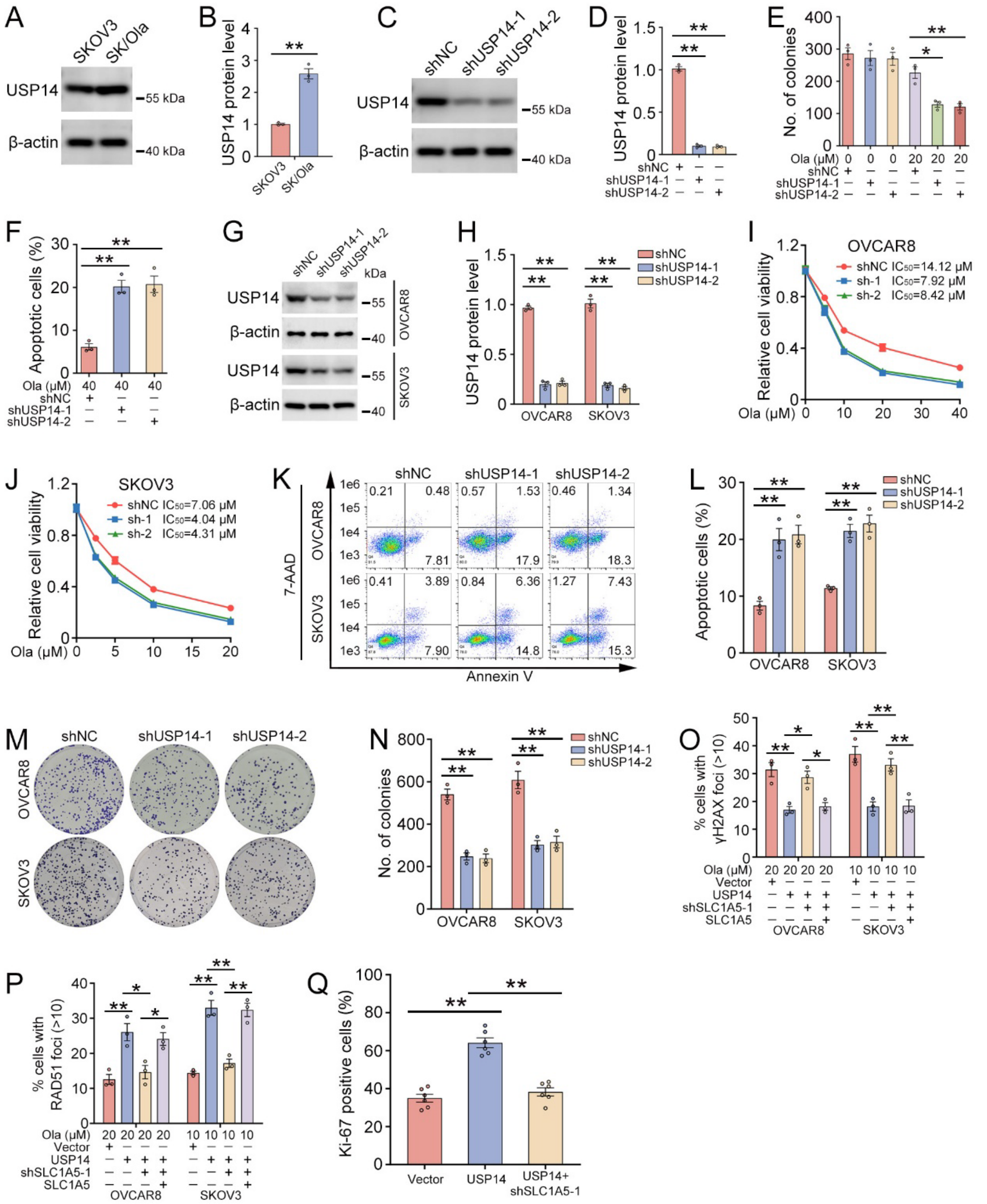

Figure S7. (A) Western blot was performed to determine USP14 and β-actin protein levels in SKOV3 and SK/Ola cells. (B) Quantification of USP14 protein levels from (A) is provided. (C) SK/Ola cells were stably transfected with pLKO.1 (shNC), USP14 shRNA 1 (shUSP14-1), or USP14 shRNA 2 (shUSP14-2) plasmids, and knockdown efficiency was verified by western blot. (D) Quantification of USP14 protein levels from (C) is provided. (E) The number of colonies from Figure 7B is quantified. (F) The proportion of apoptotic cells from Figure 7C is quantified. (G) OVCAR8 and SKOV3 cells were stably transfected with pLKO.1 (shNC), USP14

shRNA 1 (shUSP14-1), or USP14 shRNA 2 (shUSP14-2) plasmids, and knockdown efficiency was confirmed by western blot. (H) Quantification of USP14 protein levels from (G) is provided. (I) and (J) Cell viability was assessed by MTT assay after 72 h of treatment with increasing concentrations of olaparib. (K) Apoptosis was analyzed by flow cytometry in cells treated with olaparib (OVCAR8: 20 μM; SKOV3: 10 μM) for 72 h. (L) The proportion of apoptotic cells is quantified based on the results from (K). (M) Colony formation assay was performed in cells treated with olaparib (OVCAR8: 10 μM; SKOV3: 5 μM). (N) The number of colonies from (M) is quantified. (O) and (P) Quantification of the percentage of cells with γH2AX foci (>10) and RAD51 foci (>10) in Figure 7I and J, respectively,. (Q) Quantification of the Ki-67 positive ratio in Figure 7M. Data are presented as the mean ± SEM (*p < 0.05, **p < 0.01, n = 3).

Supplementary Table S2

Correlation between SLC1A5 expression and clinicopathological characteristics in HGSOC patients

Clinicopathological features		SLC1A5 expression		
		Low expression	High expression	*p* value
Age (years)	<56	36	42	0.8262
	≥56	72	79	
FIGO stage	I–II	29	27	0.4251
	III–IV	79	94	
CA125 (U/mL)	<600	51	48	0.3900
	≥600	52	62	
Lymph node metastasis	Positive	39	54	0.3766
	Negative	30	31	
Omentum metastasis	Positive	41	63	0.0163
	Negative	39	28	

Supplementary Table S3

The shRNA sequences targeting USP14 and SLC1A5

Gene	Sequences (5' to 3')
shNC	TTCTCCGAACGTGTCACGT
shUSP14-1	GCAGCCCTTAGAGATTTGT
shUSP14-2	GCCTCGCAGAGTTGAAATA
shSLC1A5-1	CCGGTCCTGTACCGTCCTCAA
shSLC1A5-2	TCGCTCATACTCTACCACCTA

Supplementary Table S4

The primer sequences used for qRT-PCR.

Gene	Sequences (5' to 3')
SLC1A5-F	CATCGTCTTTGGTGTGGCG
SLC1A5-R	CACAGGGGCGTACCACATG
β-actin-F	CATGTACGTTGCTATCCAGGC
β-actin-R	CTCCTTAATGTCACGCACGAT
